# *para*-Donor Effects in PyNO Push Ligands Control O–O Bond Cleavage of TPAFe(III)-Acylperoxo to High-Valent Fe(IV)=O or Fe(IV)=O Radical Cation Species

**DOI:** 10.1021/acs.inorgchem.5c05784

**Published:** 2026-02-28

**Authors:** Chang-Quan Wu, Po-Chun Yang, Tzu-Hsien Tseng, Wan-Qin Zeng, Hui-Ling Cheng, Tao-Hsien Liu, Chung-Wei Li, I-Chung Lu, Peter Ping-Yu Chen

**Affiliations:** Department of Chemistry, 34916 National Chung Hsing University, Taichung 40227, Taiwan (R.O.C)

## Abstract

Selective O–O bond cleavage in nonheme Fe­(III)–OOR complexes supported by tetradentate N_4_ ligands has long been challenging. Here we show that *para*-donor-substituted pyridine *N*-oxides (PyNOs) act as powerful external push ligands that direct Fe­(III)–acylperoxo intermediates toward homolysis or heterolysis. Treatment of the μ-oxo dimer [(TPA)­(H_2_O)­Fe^III^(μ-O)­Fe^III^(H_2_O)­(TPA)]^4+^ (**1**) with X-PyNO (X = H, OMe, NMe_2_) and *m*CPBA at −40 °C generates the elusive acylperoxo species [(TPA)­(X-PyNO)­Fe^III^–OOC­(Ar)]^2+^ (**2** ^**X**^). PyNO and OMe-PyNO promote homolytic O–O cleavage to afford high yields of the *S* = 1 Fe­(IV)=O species, [(TPA)­(PyNO)­Fe^IV^=O]^2+^ (**3**), corroborated by a λ_max_ at 727 nm and low-temperature ^1^H NMR data supporting PyNO binding. In contrast, strongly donating NMe_2_-PyNO enforces heterolysis, producing a short-lived *S* = 1/2 oxoiron intermediate with near-isotropic EPR (*g* = 2.024, 2.011, 1.992) and intense UV–vis absorption at 586 and 637 nm. DFT analysis reveals antiferromagnetic coupling between an Fe^IV^=O unit and the NMe_2_-PyNO**·** ^+^ ligand, defining this species as an Fe­(IV)=O–PyNO**·** ^+^ radical cation (**4**). Complex **4** oxidizes alkanes with C–H BDEs up to 99 kcal·mol^–1^ and exhibits a large KIE = 7.9 (toluene/toluene-*d*_8_), establishing ligand-controlled access to reactive oxoiron states for C–H activation.

## Graphical abstract

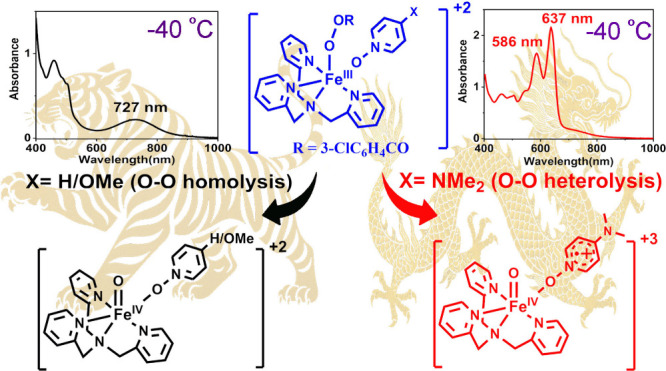

## Introduction

High-valent, mononuclear, nonheme Fe­(V)=O complexes are widely proposed as active intermediates in nonheme enzymes, by analogy to the Fe­(IV)=O heme porphyrin π-cation radical (Compound I) that mediates efficient oxygen-atom transfer (OAT).^1–16^ Yet such highly reactive species and their definitive spectroscopic characterization remain formidable challenges.^9–16^ A benchmark case is the anionic [(TAML)­FeV­(O)]^−^ (TAML = tetraamido macrocyclic ligand), which has been comprehensively characterized by UV–vis, electron paramagnetic resonance (EPR), Mössbauer spectroscopy, X-ray absorption spectroscopy (including EXAFS), and electrospray ionization mass spectrometry (ESI-MS), for example, upon oxidation of an Fe­(III)–TAML precursor with *m*-chloroperbenzoic acid (*m*CPBA) at −60 °C.^9,15^ These observations underscore how an anionic macrocycle stabilizes the Fe­(V) oxidation state and promotes O–O bond heterolysis from Fe­(III)–OOR precursors. In neutral aminopyridine platforms such as TPA and TPA* (Scheme 1), reactions of TPA/TPA*-supported Fe­(II) complexes *m*CPBA preferentially afford Fe­(III)–acylperoxo intermediates, which often accumulate as the dominant spectroscopically observable species.^17,18^ Although high-valent iron–oxo species have been implicated under related conditions and, in some cases, detected by highly sensitive techniques such as EPR, for which concentration sensitivities down to ∼10–100 μM have been reported for iron species,^19–21^ or mass spectrometry,^14^ their direct optical identification by UV–vis spectroscopy has remained elusive in neutral TPA/TPA* systems. In addition, reactions of Fe­(II) complexes with hydrogen peroxide typically generate Fe­(III)–OOH intermediates, for which O–O bond heterolysis can be promoted by proton-transfer networks involving iron-bound water molecules or carboxylate ligands, leading to high-valent iron–oxo species that are most commonly detected by EPR spectroscopy.^16,17,22–26^ However, in these systems, the resulting Fe­(V)=O species are generally short-lived and rarely exhibit distinct or persistent UV–vis absorption features.

**Scheme 1:**
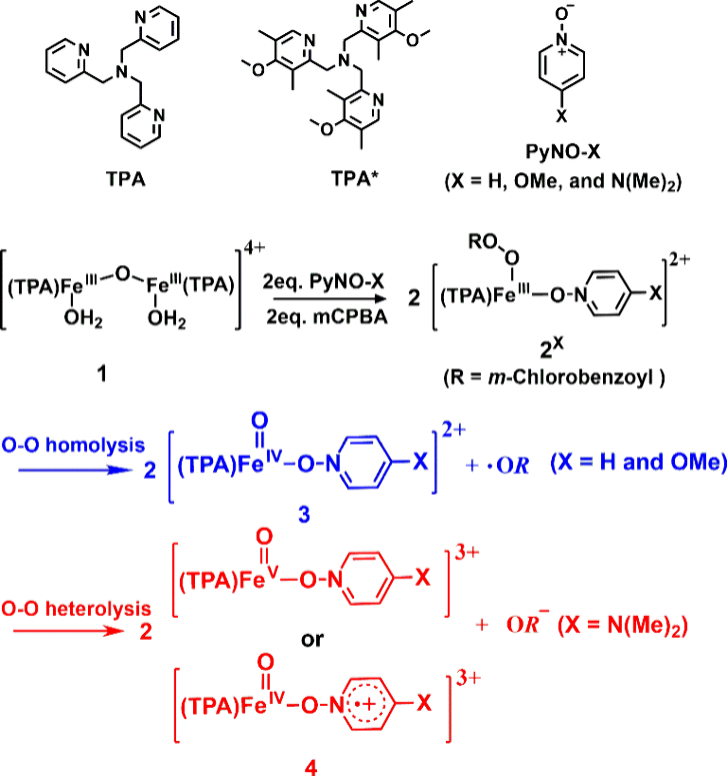
Proposed Formations of Fe­(IV)=O and Fe­(IV)=O Cationic Radicals

Importantly, the absence of definitive UV–vis signatures could result from the concentration of the resulting Fe­(V)=O species falling below the detection limit, or when significant spectral overlap occurs between Fe­(III)–OOH/Fe­(III)–OOR intermediates and nascent Fe­(V)=O species, UV–vis spectroscopy alone becomes inadequate for reliably assessing the efficiency of O–O bond heterolysis leading to Fe­(V)–oxo or closely related high-valent analogues. These intrinsic limitations hinder direct optical interrogation of O–O bond cleavage pathways under *m*CPBA-based neutral conditions.

Within this context, strategies that can electronically bias Fe­(III)–acylperoxo intermediates toward controlled O–O bond cleavage, while simultaneously enabling spectroscopic discrimination of the resulting high-valent iron–oxo species, remain highly desirable. The present study demonstrates that *para*-donor-substituted pyridine *N*-oxide ligands act as external electronic push ligands that modulate O–O bond cleavage pathways of TPA-supported Fe­(III)–acylperoxo intermediates, allowing direct spectroscopic observation of distinct high-valent iron–oxo via O–O bond heterolysis.

In this study, we initiated our approach with the mononuclear complex [TPAFe­(III)]^3+^, simultaneously introducing pyridine-*N*-oxide as an external push ligand along with *m*CPBA to generate [(TPA)­(X-PyNO)­Fe­(III)–OOR]^3+^(**2** ^**X**^), X = H, OMe, and NMe_2_. This was achieved by first allowing these pyridine *N*-oxides to react with the dinuclear complex [(TPA)­(H_2_O)­Fe^III^(μ-O)­Fe^III^(H_2_O)­(TPA)]^4+^ (**1**), followed by the addition of *m*CPBA, as illustrated in Scheme 1.

## Results and Discussion

Herein, the MeCN solution of **1** (4.86 × 10^–1^ mM) was reacted with 2 equiv of PyNO for 10 min at room temperature. The EPR and ^1^H NMR spectra reveal an intermediate-spin monomer Fe­(III) species with PyNO coordination (Figures S1 and S2). Upon the addition of 2 equiv of *m*CPBA in the above solution at −40 °C, an instantaneous change in the absorption spectrum was observed within 30 s, revealing the character of Fe­(IV)=O species (**3**) at 726 nm as shown in Figure 1a and 1b for X-PyNO, X = H and OMe, respectively. The well-resolved ^1^H NMR data obtained at −40 °C provide compelling evidence for the formation of highly concentrated Fe­(IV)=O species, exhibiting a pattern and chemical shifts that closely match those of the well-characterized [(N_4_Py)­Fe­(IV)=O]^2+^ and [(TPA)­Fe­(IV)­O­(CH_3_CN)]^2+^.^27,28^ Furthermore, the coordination of PyNO to the mononuclear Fe­(IV)=O center was unambiguously confirmed by a significant downfield shift of 64.7 ppm, as observed using pyridine-*d*_5_-*N*-oxide, as shown in Figure 2(b).

**Figure 1:**
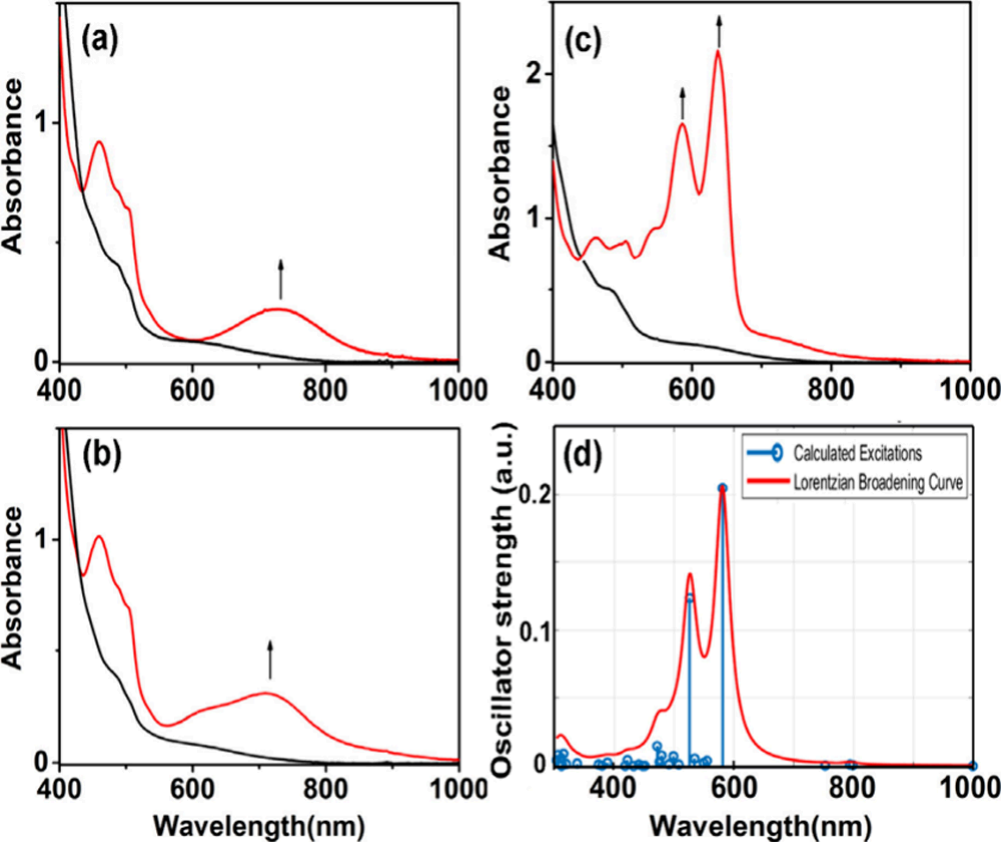
UV–vis spectral changes of **1** (4.86 × 10^–1^ mM; black line) to the product (red line) under different conditions: (a) with 2 equiv of pyNO and 2 equiv of *m*CPBA in CH_3_CN (−40 °C); (b) with 2 equiv of pyNO-OMe and 2 equiv of *m*CPBA in CH_3_CN (−40 °C); and (c) with 2 equiv of NMe_2_-PyNO and 2 equiv of *m*CPBA in CH_3_CN (−40 °C). (d) TD-DFT-calculated absorption spectra for the optimized structure of [(TPA)­(NMe_2_-PyNO**·** ^+^)­Fe­(IV)=O)]^3+^(**4**), including 50 excited states.

**Figure 2:**
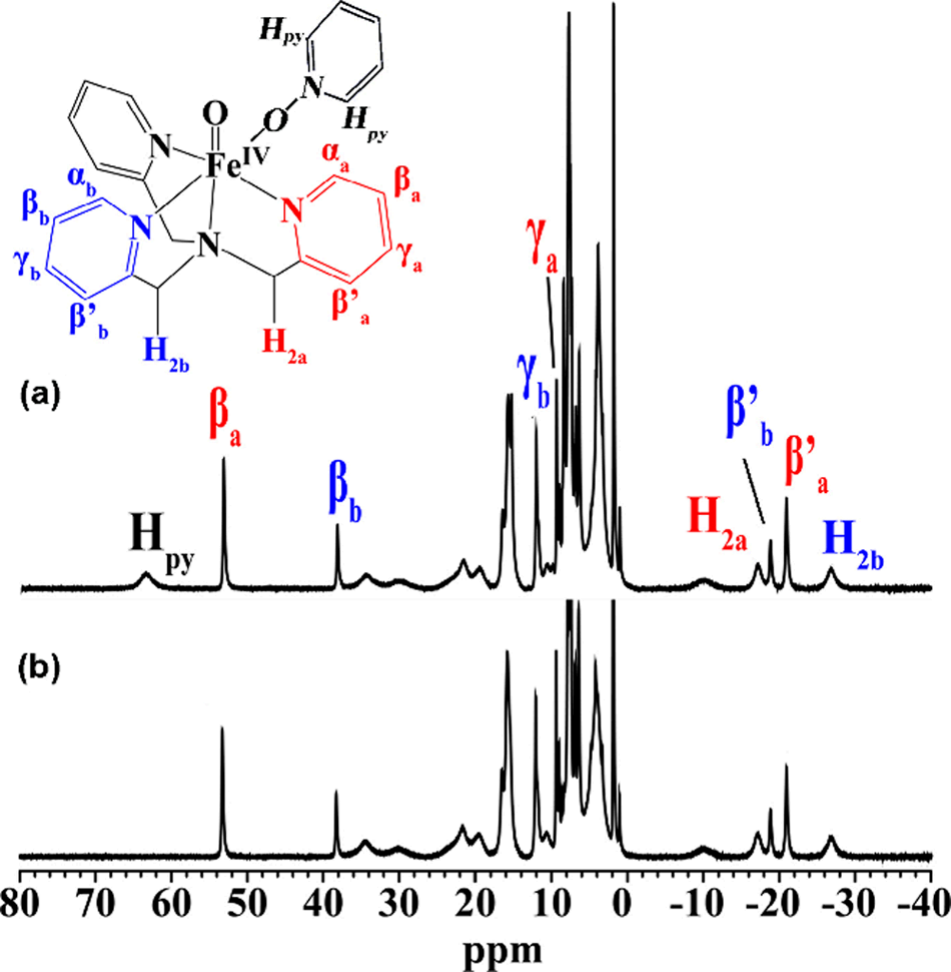
600 MHz ^1^H NMR spectra (−40 °C) showing the formation of the mononuclear iron­(IV)=O species upon the addition of (a) pyridine-*N*-oxide or (b) pyridine-*d*_5_-*N*-oxide to the CD_3_CN solution of **1** and *m*CPBA.

Notably, the ^1^H NMR spectra of these complexes are virtually identical to those observed when PyNO is introduced into the reaction mixture of [TPAFe­(II)­(CH_3_CN)_2_]^2+^ with 1.5 equiv of *t*-BuOOH (Figure S3). This striking similarity indicates that PyNO ligation significantly promotes the homolysis of the Fe­(III)-OO*t*Bu bond, as demonstrated by UV–vis spectral changes previously reported by Que et al.^23^ Comparable spectral data were obtained with OMe-PyNO under the same reaction conditions (Figure 1b). These findings underscore that while both PyNO and OMe-PyNO effectively coordinate to the mononuclear iron center, neither is sufficient to drive O–O bond heterolysis of Fe­(III)–OOR (R = 3-ClC_6_H_4_CO). However, they markedly accelerate the homolytic cleavage of the O–O bond.

According to the UV–vis and ^1^H NMR characteristic absorption of [(TPA)­PyNO-Fe^IV^=O]^2+^, a near 75% yield was shown for such conversion from dinuclear Fe^III^–O–Fe^III^ species (**1**). While the formation of Fe­(V)=O is not observed, formation of a mononuclear Fe­(III)–acylperoxo complex is feasible by utilizing dinuclear Fe^III^–O–Fe^III^ species in conjunction with PyNO.

Upon replacing the strongest electron-donating ligand, NMe_2_-PyNO, in the same reaction at −40 °C, a completely distinct species from Fe­(IV)=O rapidly forms within 30 s, as depicted in Figure 1(c). This new species displays two intense absorption peaks at 586 and 637 nm, closely mirroring the spectral features of [(TAML)­Fe­(V)=O]^−1^. The intermediate exhibits significant instability at −40 °C. The decline rates of these two absorbances were fitted with a simple first-order kinetic equation affording identical rate constants (*k*_obs_ = 1.71 × 10^–3^ s^–1^) at −40 °C (Figure S4). In addition, ^1^H NMR detected no Fe­(IV)=O species for this reaction.

In the cold-spray ionization mass spectrum (CSI-MS) of the above reaction, a trace amount of ion at *m*/*z* of 804.2 was also found to be consistent with the elusive [(TPA)­(NMe_2_-PyNO)­Fe­(O)­(*m*-CBA)­(OTf)]^+^, **4** (calculated *m*/*z* of 804.1). This species agrees with either Fe­(V)=O or Fe­(IV)=O ligand radical, as evidenced by the comparison with the simulated spectrum in Figure 3(b). Treating the above solution with labeled water (H_2_^18^O) induces the peak at *m*/*z* 803.9 to shift to *m*/*z* 805.9 (M + 2). This accounts for the incorporation of one ^18^O atom within the structure. In addition, two prominent ions at *m*/*z* 656.17 and *m*/*z* 788.17 were observed, respectively, whose mass and isotope distribution pattern correspond to mononuclear iron­(III) complexes, [(TPA)­(NMe_2_-PyNO)­Fe^III^(OH)­(*m*-CBA)]^+^ (*m*-CBA = *m*-chlorobenzoate) and [(TPA)­(NMe_2_-PyNO)­Fe^III^(*m*-CBA)­(OTf)]^+^, as shown in Figure S5. These two species potentially result from the active intermediate.

**Figure 3:**
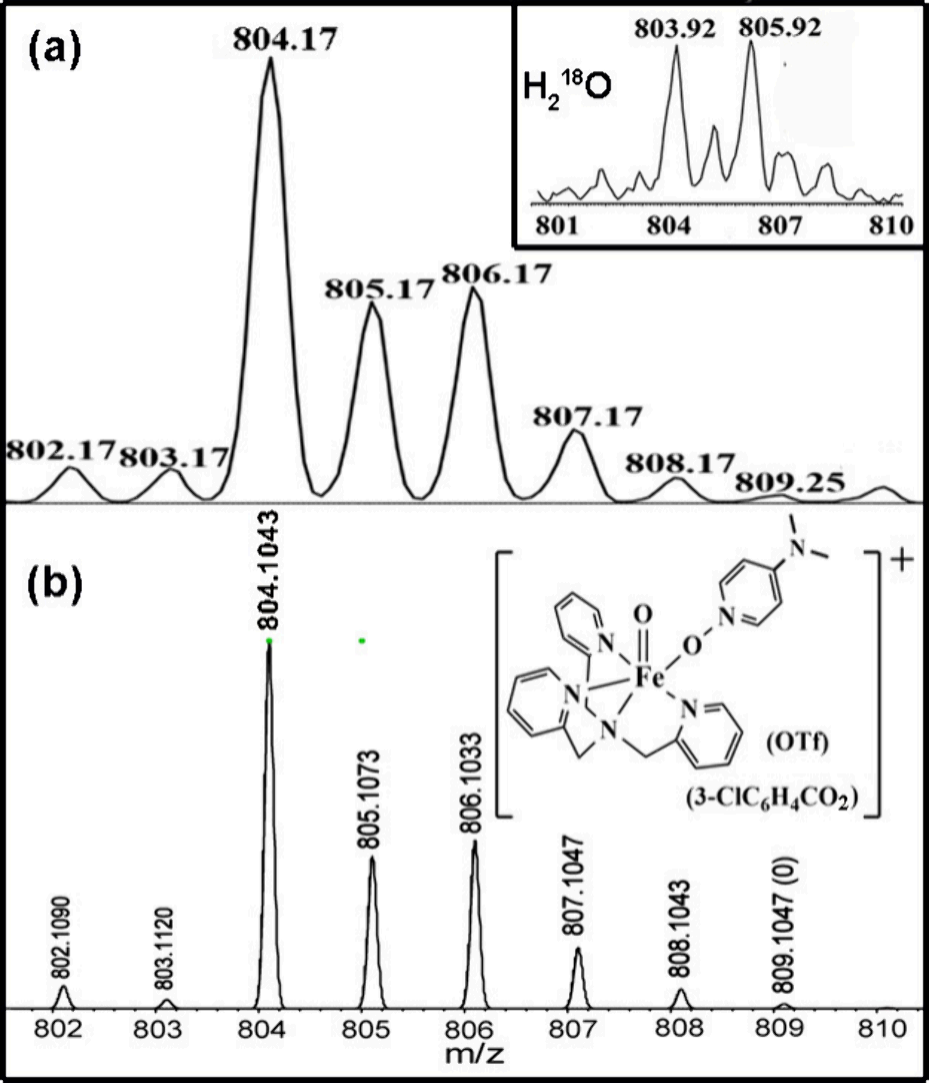
(a) CSI-MS spectrum and (b) isotope patterns simulated for [(TPA)­(NMe_2_-PyNO)­Fe­(O)­(3-chlorobenzoate)­(OTf)]^+^.

Sequential addition of two equiv of PyNO followed by two equiv of *m*CPBA remains EPR-silent, consistent with rapid formation of an Fe­(IV)=O species as evidenced by UV–vis and NMR data. By contrast, we also observe that treatment of **1** with 10 equiv of *m*CPBA affords a rhombic EPR signal at *g* = 2.51, 2.27, and 1.82 (Figure 4a), matching that reported for [(TPA)­Fe­(III)-acylperoxo]^2+^ generated from [Fe­(II)­(TPA)­(CH_3_CN)_2_]^2+^ with 20 equiv of *m*CPBA.^18^

**Figure 4:**
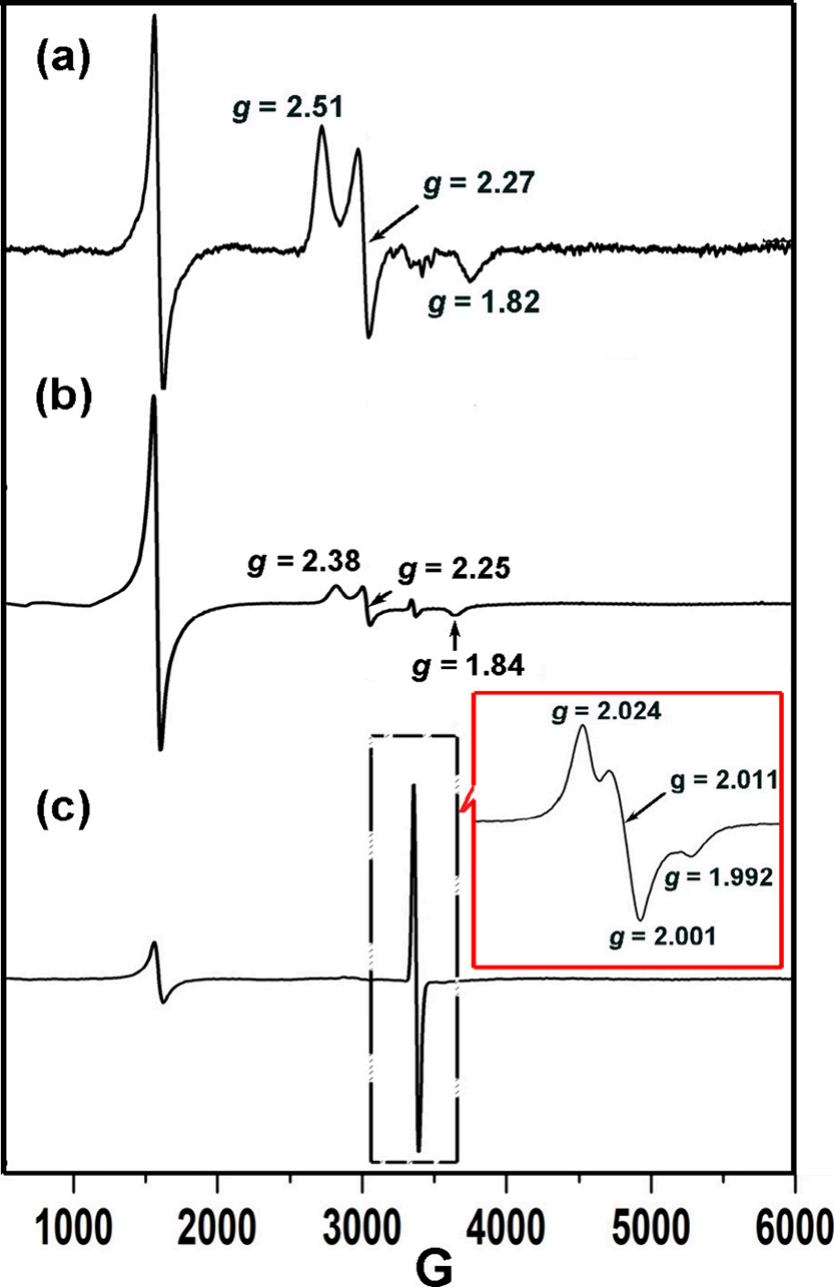
X-band EPR spectra of the Fe­(III)–O–Fe­(III) TPA dimer (**1**) in CH_3_CN at −40 °C after reacting with (a) 10 equiv of *m*CPBA, (b) 10 equiv of *m*CPBA with 5 equiv of PyNO, and (c) 2 equiv of NMe_2_-PyNO with 2 equiv of *m*CPBA. Inset: magnified region recollected using a narrow-field scan (500 G window) centered at 3410 G, corresponding to the area indicated by the dashed box. EPR conditions: *T* = 77 K; microwave power, 0.2 mW; modulation amplitude, 20 G.

Subsequent addition of 5 equiv of PyNO yields a second, transient species with *g* = 2.38, 2.25, and 1.84 (Figure 4b), diagnostic of a low-spin (S = 1/2) system and consistent with assignment to the putative PyNO-Fe­(III)-acylperoxo intermediate (**2** ^**H**^) immediately. Pre-equilibrating **1** with PyNO, followed by *m*CPBA, reproduces the same *g* = 2.38, 2.25, 1.84 signal, indicating that formation of **2** ^**H**^ is independent of reagent order. Thus, either excess *m*CPBA or PyNO alone converts the μ-oxo-bridged dimer **1** into mononuclear species. Additionally, the ^1^H NMR and the X-band EPR spectra of a reaction mixture of **1** with two equiv of NMe_2_-PyNO reveal the formation of mononuclear intermediate-spin iron­(III) species (Figure S6), mirroring the behavior observed with PyNO. Upon subsequent addition of two equiv of *m*CPBA at – 40 °C, the 77 K EPR spectrum evolves within 2 min from a *g* ≈ 4.23 feature to a strong, near-isotropic signal (*g* = 2.024, 2.011, 1.992), as shown in the inset of Figure 4(c). Time-course freezing of aliquots reveals a decay pattern with a rate constant of *k* = 1.84 × 10^–3^ s^–1^, closely mirroring the UV–vis spectral changes. This alignment indicates that the reaction dynamics influence the EPR and UV–vis properties, reflecting the same chemical transformations (Figure S7). Notably, the spectrum is incompatible with Fe­(III)–OOR or PyNO–Fe­(III)–OOR (**2** ^**H**^) benchmarks. The disappearance of Fe­(III)–OOR features, the EPR silence expected for Fe­(IV)=O, and the emergence of a near-isotropic *S* = 1/2 signal collectively implicate formation of a higher-valent Fe–oxo center.

EPR spin counting versus Fe­(III)–OO*t*Bu (≈quantitative under 2 equiv TBHP at −40 °C) gives ∼70% yield of the near-isotropic species **4**. The EPR signature departs from that of [Fe^V^(O)­(OAc)­(L)]^2+^ (2.07, 2.01, 1.96; L = TPA/TPA*) reported by Talsi and co-workers,^16^ indicating a related yet electronically distinct Fe–oxo center. Concordant UV–vis, CSI-MS, and EPR results point to efficient O–O bond heterolysis of the Fe­(III)–OOR precursor and formation of a higher-valent Fe–oxo species (**4**). Moreover, the rapid quenching of this signal upon reaction with organic substrates further supports its identification as a reactive intermediate (vide infra).

To gain insight into the influence of NMe_2_-PyNO on the electronic structure of the active intermediate, we utilized DFT calculations employing the scalar-ZORA B3LYP/TZ2P//BP86/TZ2P and scalar-ZORA PBE0/TZ2P//BP86/TZ2P levels, along with COSMO/MeCN, as implemented in the ADF program.^29^ This approach allowed us to explore molecular orbitals and absorption spectra. Detailed computational procedures are provided in the Supporting Information.

The DFT-optimized structure of [(TPA)­NMe_2_-PyNO-Fe­(O)]^3+^ (**4**) reveals a six-coordinate iron species, and the Fe=O bond is 1.64 Å (Figure 5a). Compound **4** exhibits a highly delocalized spin-density distribution, with significant population on both the iron center (+1.33) and the terminal oxo ligand (+0.76), together with ligand-centered spin on the NMe_2_-PyNO fragment (−0.88). To place these descriptors in context, we compared the computed Fe=O bond length and Fe/O spin populations with literature-reported DFT benchmarks for representative nonheme iron–oxo species (Table S1).^9,13,30–34^ Well-established Fe­(V)=O complexes typically exhibit short Fe=O bonds (1.60–1.64 Å) and predominantly metal-centered spin density (Fe: 0.64–0.69; O: ∼0.22–0.32), whereas nonheme Fe­(IV)=O species show comparable Fe=O bond lengths but substantially greater spin delocalization onto the oxo ligand (Fe: ∼1.06–1.36; O: ∼0.73–0.87).^31–33^ In this regime, the large oxo spin population is best described as oxyl character arising from strong Fe–O covalency, where unpaired spin from both π-manifold components (d_*xz*_ and d_*yz*_) is delocalized onto the oxo ligand rather than representing a localized O• species.^33^ Additionally, the calculated Mayer bond order for the Fe–O unit in **4** is 1.97, supporting a strongly covalent multiple-bond character.

**Figure 5:**
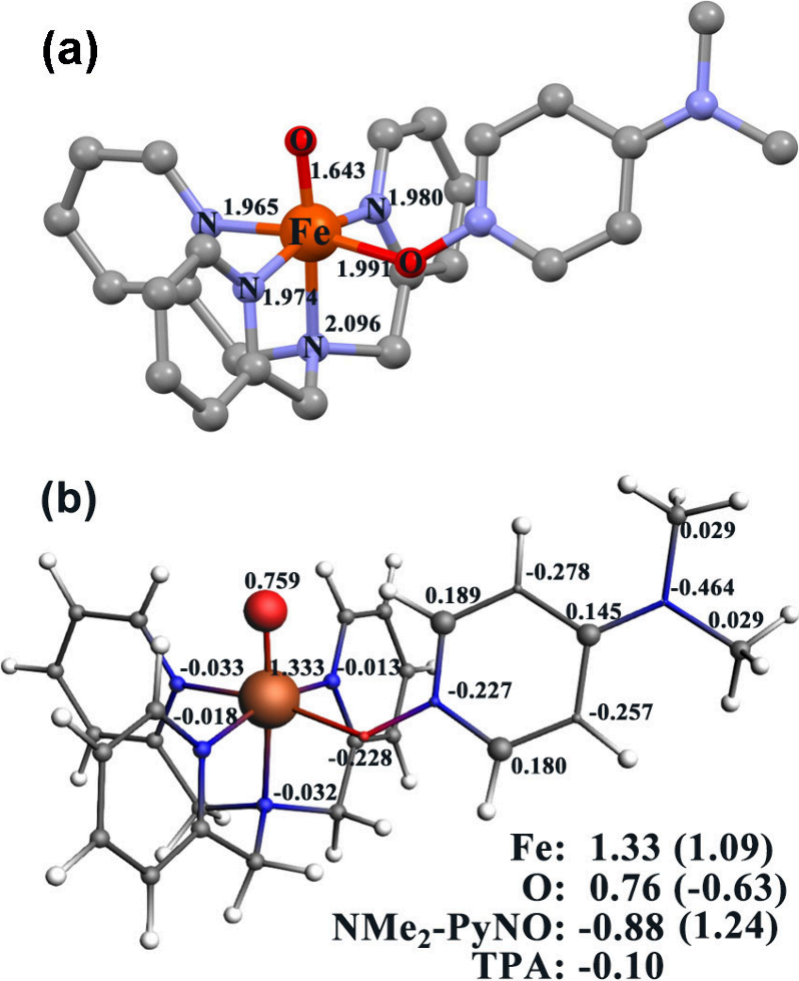
(a) DFT-optimized geometry of compound **4** obtained at the BP86/TZ2P level with implicit solvation (COSMO, MeCN). Hydrogen atoms are omitted for clarity, and selected Fe-centered bond lengths (Å) are indicated. Cartesian coordinates for all optimized structures are provided in the Supporting Information. (b) Mulliken spin-density distribution for **4** calculated at the scalar-ZORA B3LYP/TZ2P level with implicit solvation (COSMO, MeCN). Atomic spin populations with |ρ| < 0.013 are omitted, and Mulliken charges of the iron center, terminal oxo ligand, and NMe_2_-PyNO are given in parentheses.

The robustness of this assignment is further supported by results obtained using multiple computational models, as summarized in Table S2, including Fe–O bond distances, Mayer bond orders, Mulliken spin populations, and Mulliken charges. Notably, the spin-coupled iron porphyrin system [(TMP•^+^)­Fe­(IV)­(O)­(Cl)] displays closely comparable Fe–O bond lengths (∼1.65 Å) and similarly delocalized spin densities (Fe = 1.35; O = 0.82; porphyrin = −1.10 for *S* = 1/2), providing a compelling electronic-structure parallel to compound **4**.^34^ In addition, the Mulliken charges of +1.09 on Fe, −0.63 on the oxo, and +1.24 on the NMe_2_-PyNO are comparable to those reported for other nonheme Fe­(IV)=O species, such as [(TMC)­Fe^IV^O]^2+^ and [(N4PY)­Fe^IV^O]^2+^ and differ from values characteristic of [(biuret–TAML)­Fe^V^(O)]^−^ species.^35–37^ While DFT provides reliable optimized geometries, the quantitative distribution of spin density is inherently method-dependent and may therefore be subject to debate; accordingly, the electronic-structure assignment of compound **4** must be combined with the consistency of geometric, bonding, charge, spectroscopic, and literature benchmarks.

Thus, the analysis does not support an Fe­(V)=O formulation while demonstrating a complete correspondence with oxoiron­(IV)_NMe_2_-PyNO radical cation. Moreover, the qualitative spin distribution strongly correlates with the occupied α-set of HOMO–4 (Fe_*d*_*yz*_-O_*p*_*y*_), HOMO–5 (Fe_*d*_*xz*_-O_*p*_*x*_), and HOMO–6 (Fe_*d*_*xy*_), along with the occupied β-set of HOMO­(NMe_2_-PyNO_π) and HOMO–4 (Fe_*d*_*xy*_), as well as the empty LUMO (Fe_*d*_*xz*_-O_*p*_*x*_) and LUMO+1 (Fe_*d*_*xz*_-O_*p*_*x*_) (Figure 6). The molecular orbital analysis, together with the spin-density and charge distributions, indicates an electronic structure best described as an *S* = 1 Fe­(IV)=O center antiferromagnetically coupled to a NMe_2_-PyNO cation radical. An alternative electronic-structure description has been proposed for related PyNMe_3_-supported oxoiron species, in which O–O bond cleavage yields an Fe­(IV)=O center antiferromagnetically coupled to a ligand- or peroxide-derived radical rather than a purely metal-centered Fe­(V)O formulation. This framework, developed by Neese, Ye, and co-workers, is fully consistent with Fe­(IV)­O/ligand-radical description assigned to compound **4** in the present study.^38^

**Figure 6:**
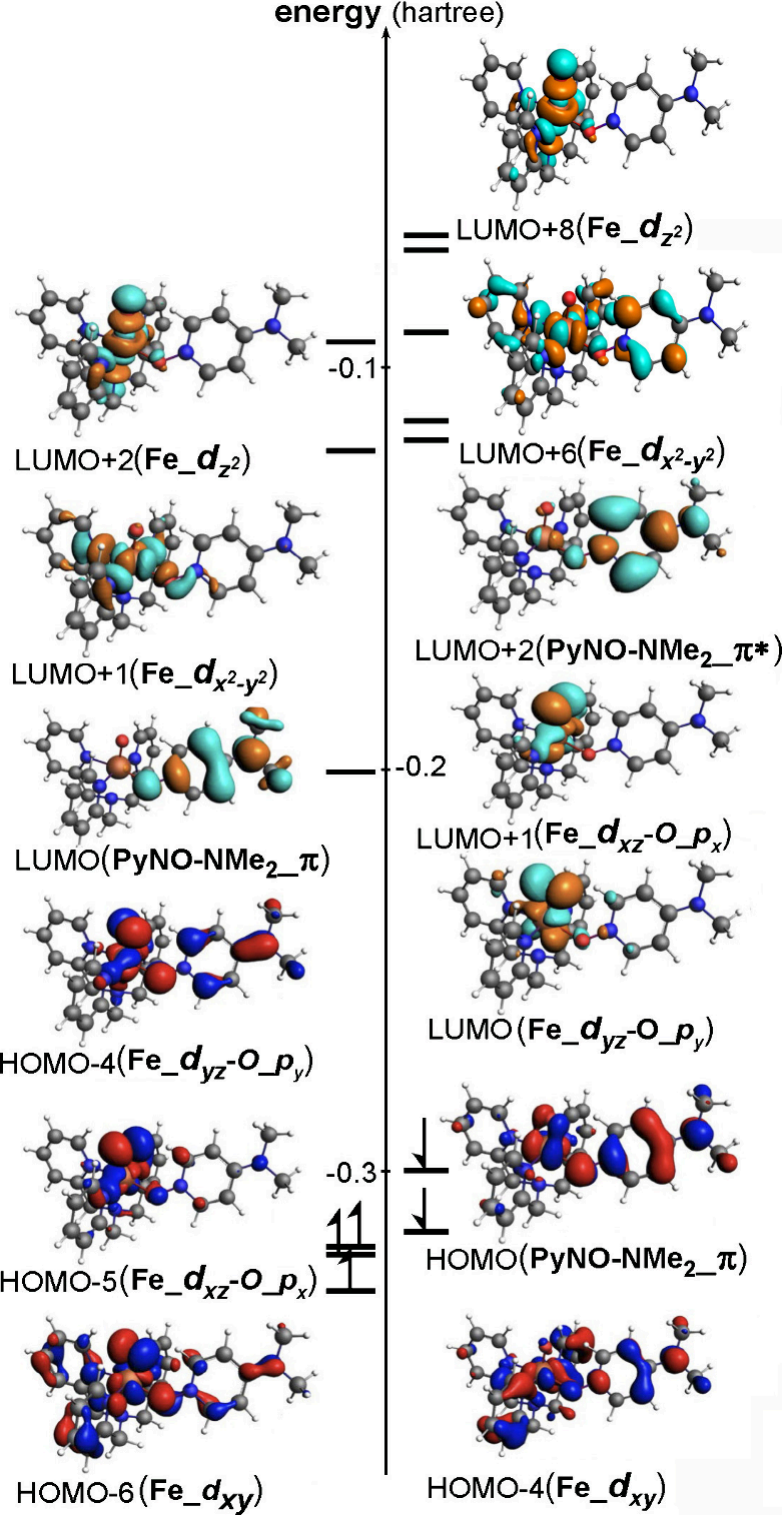
Selected molecular orbital (MO) diagram of complex **4** in its ground-state doublet configuration, obtained from DFT calculations at the COSMO (CH_3_CN) PBE0/TZVP level of theory. Additional MOs are shown in Figure S8, and the corresponding MO diagram calculated using COSMO-B3LYP/TZ2P is provided in Figure S9.

Notably, this electronic structure closely resembles the Fe­(IV)=O/ligand-radical formulation proposed by Wang et al. for the NMe_2_PDP–Fe epoxidation catalyst based on DFT calculations, in which O–O heterolysis generates a Compound-I-like nonheme Fe­(IV)–oxo species stabilized by a ligand-centered radical.^39^ This precedent further supports assignment of **4** as a ligand-radical-coupled Fe­(IV)=O manifold rather than a purely metal-centered Fe­(V)=O species. A relevant benchmark is the gas-phase IRPD-characterized [Fe^V^(O)­(OH)­(^5tips3^tpa)]^2+^ complex (*S* = 3/2) reported by Costas and co-workers, which represents a well-defined example of Fe­(V)=O chemistry supported by a TPA-type ligand framework.^40^ In contrast, intermediate **4** in the present study is generated and characterized in solution, exhibits an *S* = 1/2 EPR signature, and displays strongly delocalized spin density consistent with an Fe­(IV)=O center antiferromagnetically coupled to a ligand-centered radical. These differences underscore the sensitivity of high-valent iron–oxo electronic structures to phase and coordination environment, highlighting that closely related ligand frameworks can stabilize distinct electronic manifolds under different conditions.

TD-DFT calculations of the absorption spectrum of compound **4** at the PBE0/TZ2P level of theory, employing the COSMO/MeCN solvent model, yielded a simulated spectrum exhibiting two prominent absorption peaks at 525 and 585 nm, attributed to α-spin manifold excitations. These calculated absorption bands closely resemble the experimentally obtained absorptions at 586 and 637 nm, as shown in Figure 1(d).

The absorption peak at 525 nm is primarily associated with transitions: 28% from Fe_*d*_*yz*_-O_*p*_*y*_ (HOMO–4) to Fe_*d*_*x2‑y*2_ (LUMO+1), 16% from Fe_*d*_*yz*_-O_*p*_*y*_ to the unoccupied NMe_2_-PyNO_π (LUMO), and 11.1% from Fe_*d*_*xy*_ to LUMO. In contrast, the absorption peak at 585 nm mainly comprises transitions, with 54.2% from Fe_*d*_*yz*_-O_*p*_*y*_ (HOMO–4) to LUMO and 17% from Fe_*d*_*yz*_-O_*p*_*y*_ (HOMO–4) to Fe_*d*_*x2‑y*2_. TD-DFT calculations were also performed at the B3LYP/TZ2P level of theory, affording two major absorptions at 567 nm (75% Fe_*d*_*xy*_→ LUMO) and 661 nm (77% from­(Fe_*d*_*xz*_-O_*p*_*x*_) → LUMO (Figure S10). Thus, based on these two computed absorption spectra, the two peaks at 586 and 637 nm highly correlate with the NMe_2_-PyNO_π orbital. TD-DFT attributes the 586 and 637 nm absorptions primarily to Fe/O→PyNO–NMe_2_ π* metal-to-ligand charge-transfer (MLCT) transitions, with appreciable ligand-field (d–d) admixture involving the Fe_*d*_*x2‑y*2_ orbital. To further confirm the efficient formation of the high-valent ironoxo species by adopting the NMe_2_-PyNO ligand, it is noteworthy that another TPA ligand analogue, TPA*, was employed.

Previous reports indicated that when [(TPA*)­Fe­(II)­(CH_3_CN)_2_]^2+^ reacted with *m*CPBA, an active acylperoxo-iron­(III) species can be readily formed via UV–vis and EPR identifications.^17^ We aimed to investigate whether the addition of the electron-pushing group NMe_2_-PyNO after the generation of the mononuclear Fe­(III)-acylperoxo signal would lead to similar O–O bond heterolysis. In the absence of NMe_2_-PyNO, treatment of [(TPA*)­Fe­(II)­(CH_3_CN)_2_]^2+^ (2.21 mM, MeCN) with 5 equiv *m*CPBA at −40 °C affords only a transient 460 nm band followed by rapid decay of the Fe­(III)–acylperoxo, with no accumulation of a more reactive species (Figure 7a). By contrast, adding 2 equiv NMe_2_-PyNO to the Fe­(III)–acylperoxo at – 40 °C triggers an immediate spectral conversion to intense bands at λ_max_ = 586 and 639 nm (blue trace). This distinct UV–vis signature is in accordance with the reactive species utilizing TPA as a ligand, as Figure 7(b) demonstrates. When the low-spin EPR signal of Fe­(III)-acylperoxo species (*g* = 2.58, 2.38, 1.73) is obtained by treating 5 equiv of *m*CPBA into the MeCN solution of [TPA*Fe­(II)­(CH_3_CN)_2_]^2+^ at −40 °C,^17^ followed by the addition of 3 equiv of the NMe_2_-PyNO, a slightly rhombic signal with *g* = 2.022, 2.010, 1.990 forms as the case using NMe_2_-PyNO into the MeCN solution of **1** and *m*CPBA at −40 °C (Figure S11).

**Figure 7:**
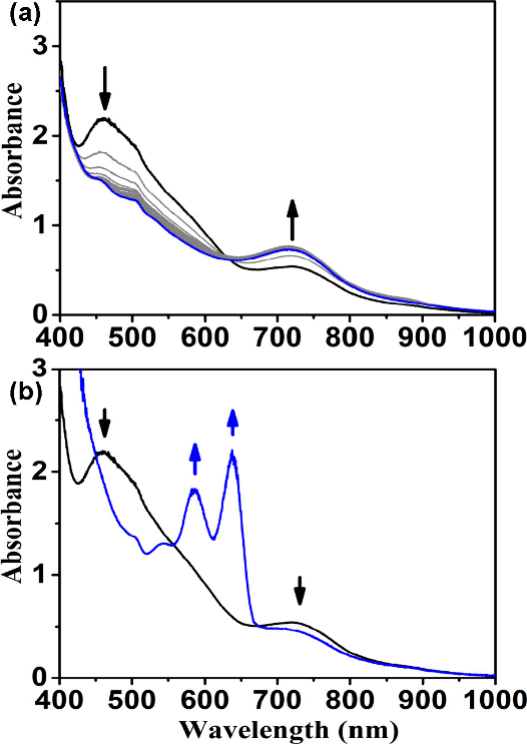
UV–vis spectral changes of [TPA*Fe­(III)–OOR]^2+^ (black line) prepared by adding 5 equiv of *m*CPBA into [TPA*Fe­(II)­(CH_3_CN)_2_]^2+^ (1 mM) (a) to the product (blue line) at −40 °C and (b) with further addition of 2 equiv of NMe_2_-PyNO at −40 °C. Moreover, the NMe_2_-PyNO-derived species is short-lived, exhibiting a first-order decay constant (*k*_obs_ = 1.72 × 10^–2^ s^–1^, Figure S12). These data indicate that NMe_2_-PyNO binding triggers efficient O–O heterolysis in [(TPA*)­(NMe_2_-PyNO)­Fe­(III)-acylperoxo]^2+^, as observed for its TPA analogue.

To investigate the activation of aliphatic C–H bonds mediated by the intermediate **4**, reactions were conducted using 1000 equiv of various substrates, including cyclohexane (C–H BDE = 99.3 kcal/mol), cyclopentane (C–H BDE = 95 kcal/mol), toluene (C–H BDE = 90 kcal/mol), and ethylbenzene (C–H BDE = 87 kcal/mol) to a MeCN solution of **4** (7.46 × 10^–1^ mM) with the exclusion of O_2_. These reactions were monitored using UV–vis spectroscopy at −40 °C. Significant optical spectral changes were observed upon adding the substrates, indicating a decrease in reaction rates following an exponential trend (see Inset of Figure 8(a)). The absorbance versus time data determined the pseudo-first-order rate constant (*k*_obs_). These values were extracted using nonlinear curve fitting based on the equation *A*_*t*_ = *A*_∞_ + (*A*_0_ – *A*_∞_)*e* ^–*k*_obst_^, where *A*_*t*_ is the absorbance at time *t*, *A*_0_ and *A*_∞_ are the initial and final absorbances at the selected wavelength. Moreover, the data reveal that the reaction rate diminishes as the C–H bond dissociation energy of the alkyl hydrocarbons increases, As shown in Figure 8(b), a linear correlation between the log *k*_2_′ values and the C–H BDE values of the substrates, which reflect the Bell–Evans–Polanyi (BEP) principle from the plot of log *k*_2_′, (the *k*_2_ values are divided by the number of equiv target C–H bonds of substrates) vs BDE.^41,42^ A significant kinetic isotope effect (KIE) of 7.9 was observed for toluene/toluene-*d*_8_ at −40 °C. This finding indicates that complex **4** is the active oxidant and that C–H bond cleavage via hydrogen-atom abstraction is the rate-determining step. At room temperature, GC–MS analysis of cyclohexane oxidation identified cyclohexanol and cyclohexanone as the products (Table 1).

**Figure 8:**
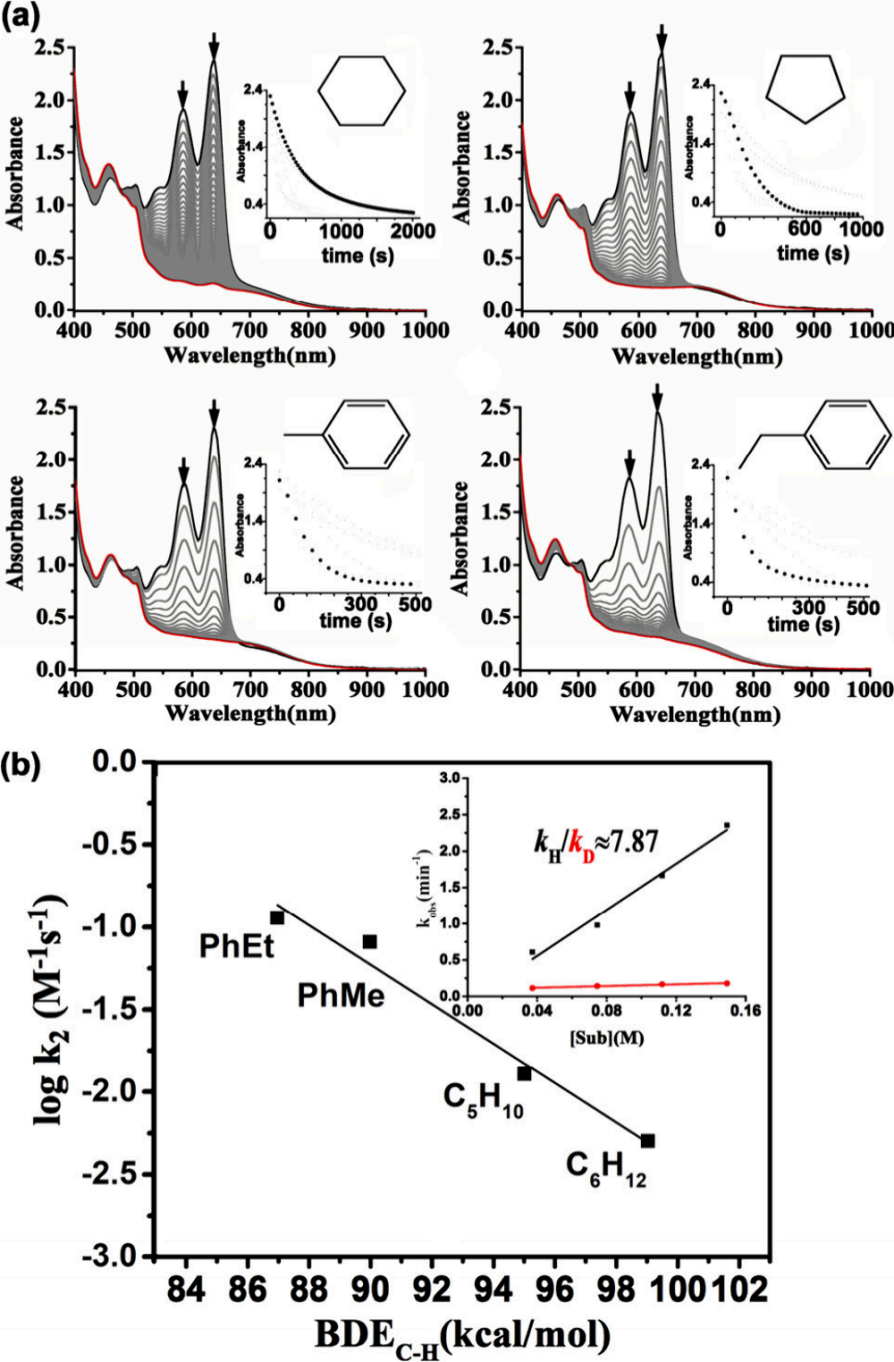
(a) UV–vis spectral changes observed upon the addition of various organic substrates (1000 equiv)—cyclohexane, cyclopentane, toluene, and ethylbenzene—into a MeCN solution of **4** at −40 °C, with spectra recorded at 30 s intervals. Inset: the absorbance vs time plot at 637 nm. (b) Relationship between log *k*_2_′ and BDE_C–H_ for different hydrocarbons in reactions with **4** at −40 °C. Inset: Plot showing *k*_obs_ (min^–1^) as a function of [toluene] (black line) and [toluene-*d*_8_] (red line), highlighting a notable kinetic isotope effect (KIE) at −40 °.

**Table 1:**
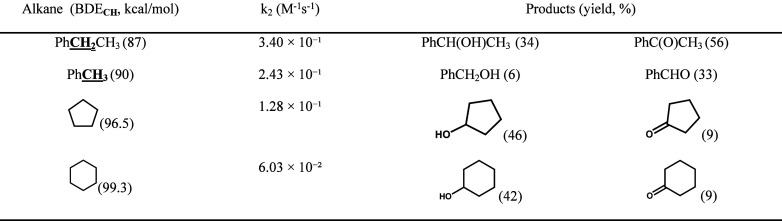
Oxidation of Hydrocarbons by the *In Situ*-Generated Intermediate **4**: Products and Reaction Kinetics^a^

a Yields (%) are calculated based on the amount of complex (**1**). Reaction conditions: substrates (9 mmol), NMe_2_-PyNO (0.006 mmol), **1** (0.003 mmol), *m*CPBA (0.006 mmol), MeCN (total volume 3.0 mL), at RT for 30 min.

Based on the amount of **1**, the reaction yield was 51% with an alcohol-to-ketone ratio of 4.7:1. Substrates bearing weaker C–H bonds, including cyclopentane, toluene, and ethylbenzene, underwent oxidation with reaction rates and products summarized in Table 1. As such, complex **4** represents an active nonheme iron species capable of mediating C–H bond oxidation under mild conditions, offering valuable mechanistic parallels to enzymatic oxidants and underscoring its potential utility in developing catalytic C–H functionalization platforms.

## Conclusions

In this study, we have demonstrated the efficient generation of high-valent iron-oxo intermediates through O–O bond cleavage of iron­(III)-acylperoxo species facilitated by pyridine *N*-oxide (PyNO) and its derivatives. The coordination of these electron-donating ligands was shown to accelerate the homolytic cleavage of Fe­(III)­O–OR, yielding Fe­(IV)=O and the heterolytic cleavage of the Fe­(III)­O–OR bond, forming a transient Fe­(IV)-oxo radical cation species. These intermediates were characterized by UV–vis, EPR, CSI-MS, and ^1^H NMR spectroscopy, supported by DFT calculations, which revealed their electronic structure and reactivity. The introduction of NMe_2_-PyNO enabled the formation of a highly reactive species with spectral features analogous to Fe­(V)=O complexes. However, computational and experimental evidence suggested an Fe­(IV)=O radical cation. This intermediate displayed pronounced reactivity toward aliphatic C–H bonds, exhibiting a clear correlation with bond dissociation energy and significant kinetic isotope effects, further confirming hydrogen abstraction as the rate-determining step.

These findings highlight the crucial role of strong electron-pushing ligands in promoting O–O bond cleavage and stabilizing high-valent intermediates. Specifically, the introduction of push ligands into the dinuclear Fe­(III)–O–Fe­(III) system, in the presence of *m*CPBA, facilitates the formation of highly concentrated and reactive [TPAFe­(IV)=O]^2+^ radical species. Notably, this strategy can also be successfully applied to [TPA*Fe­(III)–OOR]^2+^, further demonstrating its versatility in generating high-valent iron-oxo intermediates. These insights provide a foundation for the rational design of nonheme iron-based catalysts for oxidative transformations, highlighting the potential of these reactive species in selective C–H bond activation and advancing the field of bioinspired oxidation chemistry.

## Experimental Section

### Chemicals

All air- or moisture-sensitive reactions were carried out in dried glassware under a nitrogen atmosphere. Commercial *m*CPBA (10.0 g, 75%) was dissolved in Et_2_O (70 mL) and washed three times with a pH 7.5 buffer solution (50 mL). The organic layer was dried over MgSO_4_, and the solvent was carefully evaporated to afford purified *m*CPBA as a dry, white solid.^43^ Purity (>95%) was verified by ^1^H NMR and iodometric assay. Purified *m*CPBA was stored at −20 °C under N_2_ and used within 2 weeks. Deuterated solvents, including acetonitrile-*d*_3_ (CD_3_CN, 99.8 atom % D), acetone-*d*_6_ (C_3_D_6_O, 99.9 atom % D), and dichloromethane-*d*_2_ (CD_2_Cl_2_, 99.5 atom % D), were purchased from Sigma–Aldrich (Steinheim, Germany). *tert*-Butyl hydroperoxide (*t*BuOOH, 70% in H_2_O) was purchased from Aldrich (WI, USA). Dried solvents were distilled before use: acetonitrile was purchased from Sigma–Aldrich. **Caution:** *m*CPBA is known to be explosive in its very pure states and therefore should be handled with extreme care.

### Materials

The complexes [Fe^II^(TPA)­(CH_3_CN)_2_]^2+^ (TPA = tris­(2-pyridylmethyl)­amine), [Fe^II^(TPA*)­(CH_3_CN)_2_]^2+^ (TPA = tris­((4-methoxy-3,5-dimethylpyridin-2-yl)­methyl)­amine), and [(TPA)­(H_2_O)­Fe^III^(μ-O)­Fe^III^(H_2_O)­(TPA)]­(OTf)_4_ (**1**) were synthesized following previously reported procedures.^27,44,45^ Crystalline samples of [Fe^II^(TPA)­(CH_3_CN)_2_]^2+^, [Fe^II^(TPA*)­(CH_3_CN)_2_]^2+^, and complex **1** were used for spectroscopic experiments, and their crystal structures are provided in Figures S13, S14, and S15. For time-resolved NMR studies, the first NMR spectrum at *t* = 1 min was collected 1 min after mixing the reactants at −40 °C. Minor delays due to NMR setup were minimized and carefully controlled within 10–30 s, with all spectra recorded within 1 min of reaction initiation.

### Instrumentation

UV–vis spectra and all kinetic experiments were performed on a CARY60 UV–vis spectrophotometer (Agilent Technologies) and a USP-203-B Unisoku cryostat, which permits monitoring of the temperature of the experiments from 193 to 373 K. All UV–vis spectra were measured using a four-side transparent quartz cuvette with a screw cap (path length: 10 mm). Product analyses were conducted using GC measurements on an Agilent Technologies 7820A gas chromatograph equipped with a 16-sample automatic liquid sampler and a flame ionization detector (FID). EPR spectra were recorded on a Bruker EPR 300E spectrometer at approximately 77 K with frozen solutions (0.2 mL) introduced into a quartz Dewar (3 mm inner diameter) cooled with liquid nitrogen. The EPR instrument was operated at a microwave frequency of ∼9.50 GHz, microwave power 0.2 mW, modulation frequency 100 kHz, and modulation amplitude 20 G for rhombic species and 1–3 G for isotropic species. The products were purified by flash chromatography with silica gel (0.063–0.2 mm). ^1^H NMR spectra were recorded on a JEOL ECX-400 in CDCl_3_ or CD_3_CN with tetramethylsilane as an internal standard. All low-temperature ^1^H NMR spectra were collected on an Agilent 600 MHz spectrometer at variable temperatures and processed using Agilent VnmrJ software (version 4.2, Agilent Technologies, Santa Clara, USA).

Unstable iron-oxo intermediates were characterized using a cold-spray ionization mass spectrometry (CSI-MS) system. The system consisted of a home-built CSI source coupled to a linear ion trap mass spectrometer (LTQ XL, Thermo Fisher Scientific). The sample transport line was constructed from a dual-layered tube to control the sample temperature. The gap between the layers was continuously purged with cold nitrogen gas to maintain the line at −5 °C prior to ionization. The sampling line was rinsed three times (or more, depending on the quality of the spectrum) with MeCN. An aliquot of the iron intermediate solution was injected into the CSI source. A spray voltage of 4.2 kV and a tube lens voltage of 40 V were applied. The temperature of the ion transfer tube was set to 205 °C to remove solvent from the charged droplet before mass analysis.

### Hydrocarbon Oxidation

Oxidation reactions were carried out by generating the reactive intermediate **4** *in situ* from complex **1** (3.0 μmol) and NMe_2_-PyNO (6.0 μmol) upon addition of *m*CPBA (6.0 μmol) in CH_3_CN (3.0 mL) at −40 °C under an inert atmosphere. After formation of **4**, hydrocarbon substrates (9.0 mmol) were introduced, and the reaction mixture was allowed to warm to room temperature and stirred for 30 min. Reaction products were analyzed by GC–MS using calibrated response factors. Product yields are reported relative to the initial amount of iron precursor **1**, which serves as an upper bound for the maximum amount of reactive intermediate that can be generated.

### Computational Methods

All DFT calculations were carried out using the Amsterdam Density Functional (ADF) program.^29,46^ Scalar relativistic effects were included via the zero-order regular approximation (ZORA). Geometry optimizations were performed at the scalar-ZORA BP86/TZ2P level using a triple-ζ basis set with two polarization functions (TZ2P) for all atoms. Solvent effects were included using the COSMO model with MeCN (ε = 35.7) as the solvent. All geometries were optimized without symmetry constraints. Single-point energy calculations and electronic structure analyses were conducted using scalar-ZORA B3LYP/TZ2P and scalar-ZORA PBE0/TZ2P levels on BP86/TZ2P-optimized geometries.^46–51^ Spin density distributions were computed as the difference between α- and β-spin densities and visualized using ADFview. Frontier molecular orbital analysis was performed using both PBE0 and B3LYP functionals. Time-dependent DFT (TD-DFT) calculations were performed at the scalar-ZORA PBE0/TZ2P and B3LYP/TZ2P levels using the COSMO/MeCN model to simulate UV–vis absorption spectra.

## Supplementary Material



## Notes

EPR spectra, ^1^H NMR spectra, CSI-MS spectra, UV–vis spectra, Cartesian coordinates, FMOs of species **4**, and supplementary tables and figures (PDF)

## References

[1] de
Montellano P. R. O. (2010). Hydrocarbon Hydroxylation by Cytochrome P450 Enzymes. Chem. Rev..

[2] Denisov I. G., Makris T. M., Sligar S. G., Schlichting I. (2005). Structure
and chemistry of cytochrome P450. Chem. Rev..

[3] Meunier B., de Visser S. P., Shaik S. (2004). Mechanism of oxidation reactions
catalyzed by cytochrome P450 enzymes. Chem.
Rev..

[4] Poulos T. L. (2014). Heme Enzyme
Structure and Function. Chem. Rev..

[5] Kal S., Xu S. N., Que L. R. (2020). Bio-inspired
Nonheme Iron Oxidation
Catalysis: Involvement of Oxoiron­(V) Oxidants in Cleaving Strong C-H
Bonds. Angew. Chem.-Int. Ed..

[6] Zhang X. P., Chandra A., Lee Y. M., Cao R., Ray K., Nam W. (2021). Transition metal-mediated O-O bond
formation and activation in chemistry
and biology. Chem. Soc. Rev..

[7] Guo M., Corona T., Ray K., Nam W. (2019). Heme and Nonheme High-Valent
Iron and Manganese Oxo Cores in Biological and Abiological Oxidation
Reactions. ACS Cent. Sci..

[8] Chen K., Que L. (2001). Stereospecific alkane
hydroxylation by non-heme iron catalysts: Mechanistic
evidence for an Fe^V^=O active species. J. Am. Chem. Soc..

[9] de
Oliveira F. T., Chanda A., Banerjee D., Shan X. P., Mondal S., Que L., Bominaar E. L., Münck E., Collins T. J. (2007). Chemical and spectroscopic evidence for an Fe^V^-Oxo complex. Science.

[10] McDonald A. R., Que L. (2013). High-valent nonheme
iron-oxo complexes: Synthesis, structure, and
spectroscopy. Coord. Chem. Rev..

[11] Oloo W. N., Que L. (2015). Bioinspired Nonheme
Iron Catalysts for C-H and C = C Bond Oxidation:
Insights into the Nature of the Metal-Based Oxidants. Acc. Chem. Res..

[12] Fan R. X., Serrano-Plana J., Oloo W. N., Draksharapu A., Delgado-Pinar E., Company A., Martin-Diaconescu V., Borrell M., Lloret-Fillol J., García-España E., Guo Y. S., Bominaar E. L., Que L., Costas M., Münck E. (2018). Spectroscopic and DFT Characterization of a Highly
Reactive Nonheme Fe^v^-Oxo Intermediate. J. Am. Chem. Soc..

[13] Van
Heuvelen K. M., Fiedler A. T., Shan X. P., De Hont R. F., Meier K. K., Bominaar E. L., Münck E., Que L. (2012). One-electron oxidation of an oxoiron­(IV) complex to form an O = Fe^V^=NR + center. Proc. Natl. Acad. Sci.
U. S. A..

[14] Prat I., Mathieson J. S., Güell M., Ribas X., Luis J. M., Cronin L., Costas M. (2011). Observation of Fe­(V)=O using
variable-temperature mass spectrometry and its enzyme-like C-H and
C = C oxidation reactions. Nat. Chem..

[15] Ghosh M., Singh K. K., Panda C., Weitz A., Hendrich M. P., Collins T. J., Dhar B. B., Sen Gupta S. (2014). Formation
of a Room Temperature Stable Fe^V^(O) Complex: Reactivity
Toward Unactivated C-H Bonds. J. Am. Chem. Soc..

[16] Lyakin O. Y., Zima A. M., Samsonenko D. G., Bryliakov K. P., Talsi E. P. (2015). EPR Spectroscopic Detection of the
Elusive Fe^V^=O Intermediates in Selective Catalytic Oxofunctionalizations
of
Hydrocarbons Mediated by Biomimetic Ferric Complexes. ACS Catal..

[17] Oloo W. N., Meier K. K., Wang Y., Shaik S., Münck E., Que L. (2014). Identification of a low-spin acylperoxoiron­(III)
intermediate in
bio-inspired non-heme iron-catalysed oxidations. Nat. Commun..

[18] Guisado-Barrios G., Zhang Y. T., Harkins A. M., Richens D. T. (2012). Low temperature
reaction of Fe­(TPA)­(CH_3_CN)_2_
^2+^ with
excess 3-chloroperoxybenzoic acid in semi-frozen acetonitrile; EPR
detection of an acylperoxo iron­(III) adduct. Inorg. Chem. Commun..

[19] Hagen W. R. (2018). EPR spectroscopy
of complex biological iron-sulfur systems. J.
Biol. Inorg. Chem..

[20] Ding H. G., Demple B. (2000). Direct nitric oxide signal transduction
via nitrosylation
of iron-sulfur centers in the SoxR transcription activator. Proc. Natl. Acad. Sci. U. S. A..

[21] Engbers S., Guo Y. S., Klein J. (2023). A Porphyrin
Iron­(III) π-Dication
Species and its Relevance in Catalyst Design for the Umpolung of Nucleophiles. Angew. Chem.-Int. Ed..

[22] Lyakin O. Y., Bryliakov K. P., Britovsek G. J. P., Talsi E. P. (2009). EPR Spectroscopic
Trapping of the Active Species of Nonheme Iron-Catalyzed Oxidation. J. Am. Chem. Soc..

[23] Kaizer J., Costas M., Que L. (2003). A dramatic push effect on the homolysis
of Fe^III^(OOR) intermediates to form non-heme Fe^IV^=O complexes. Angew. Chem.-Int. Ed..

[24] Oloo W. N., Fielding A. J., Que L. (2013). Rate-Determining
Water-Assisted O-O
Bond Cleavage of an Fe^III^-OOH Intermediate in a Bio-inspired
Nonheme Iron-Catalyzed Oxidation. J. Am. Chem.
Soc..

[25] Oloo W. N., Banerjee R., Lipscomb J. D., Que L. (2017). Equilibrating (L)­Fe^III^-OOAc and (L)­FeV­(O) Species in Hydrocarbon
Oxidations by
Bio-lnspired Nonheme Iron Catalysts Using H_2_O_2_ and AcOH. J. Am. Chem. Soc..

[26] Lyakin O. Y., Bryliakov K. P., Talsi E. P. (2011). EPR, ^1^H and ^2^H NMR, and Reactivity
Studies of the Iron-Oxygen Intermediates in
Bioinspired Catalyst Systems. Inorg. Chem..

[27] Tseng T. H., Chen P. P. Y. (2018). A Switch from
Mechanistic Competition Mediated by a
Combination of Temperature and Concentration Effects in the Oxidation
Reaction of Fe^II^(N4Py/TPA) (OTf)_2_. Chem.-Eur. J..

[28] Klinker E. J., Kaizer J., Brennessel W. W., Woodrum N. L., Cramer C. J., Que L. (2005). Structures of nonheme oxoiron­(IV) complexes from x-ray crystallography,
NMR spectroscopy, and DFT calculations. Angew.
Chem.-Int. Ed..

[29] SCM ADF2019.3; Vrije Universiteit: Amsterdam, The Netherlands. http://www.scm.com.

[30] Kwon E., Cho K. B., Hong S., Nam W. (2014). Mechanistic
insight
into the hydroxylation of alkanes by a nonheme iron­(V)-oxo complex. Chem. Commun..

[31] Lee N. Y., Mandal D., Bae S. H., Seo M. S., Lee Y. M., Shaik S., Cho K. B., Nam W. (2017). Structure and spin
state of nonheme Fe^IV^O complexes depending on temperature:
predictive insights from DFT calculations and experiments. Chem. Sci..

[32] Jackson T. A., Rohde J. U., Seo M. S., Sastri C. V., DeHont R., Stubna A., Ohta T., Kitagawa T., Münck E., Nam W., Que L. (2008). Axial ligand effects on the geometric and electronic
structures of nonheme Oxoiron­(IV) complexes. J. Am. Chem. Soc..

[33] Kumar D., Hirao H., Que L., Shaik S. (2005). Theoretical
investigation of C-H hydroxylation by (N4Py)­Fe^IV^=O^2+^: An oxidant more powerful than p450?. J. Am. Chem. Soc..

[34] Gupta R., Li X. X., Cho K. B., Guo M., Lee Y. M., Wang Y., Fukuzumi S., Nam W. (2017). Tunneling Effect That
Changes the Reaction Pathway from Epoxidation to Hydroxylation in
the Oxidation of Cyclohexene by a Compound I Model of Cytochrome P450. J. Phys. Chem. Lett..

[35] Yang L. L., Chen X., Qu Z. X., Gao J. L. (2018). Combined
Multistate
and Kohn-Sham Density Functional Theory Studies of the Elusive Mechanism
of N-Dealkylation of *N,N*-Dimethylanilines Mediated
by the Biomimetic Nonheme Oxidant Fe^IV^(O)­(N4Py)­(ClO_4_)_2_. Front. Chem..

[36] Decker A., Clay M. D., Solomon E. I. (2006). Spectroscopy
and electronic structures
of mono- and binuclear high-valent non-heme iron-oxo systems. J. Inorg. Biochem..

[37] Singh K. K., Tiwari M. K., Ghosh M., Panda C., Weitz A., Hendrich M. P., Dhar B. B., Vanka K., Sen Gupta S. (2015). Tuning the
Reactivity of Fe^V^(O) toward C-H Bonds at Room Temperature:
Effect of Water. Inorg. Chem..

[38] Mondal B., Neese F., Bill E., Ye S. F. (2018). Electronic Structure
Contributions of Non-Herne Oxo-Iron­(V) Complexes to the Reactivity. J. Am. Chem. Soc..

[39] Wang F., Sun W., Xia C. G., Wang Y. (2017). DFT studies of the substituent effects
of dimethylamino on non-heme active oxidizing species: iron­(V)-oxo
species or iron­(IV)-oxo acetate aminopyridine cation radical species?. J. Biol. Inorg. Chem..

[40] Borrell M., Andris E., Navrátil R., Roithová J., Costas M. (2019). Characterized cis-Fe^V^(O)­(OH)
intermediate
mimics enzymatic oxidations in the gas phase. Nat. Commun..

[41] Bell R. P., Hinshelwood C. N. (1936). The Theory
of Reactions Involving Transfers. Proc. R. Soc.
London, Ser. A.

[42] Evans M. G., Polanyi M. (1938). Inertia and Driving Force of Chemical Reactions. Trans. Faraday Soc..

[43] Aggarwal V. K., Gultekin Z., Grainger R. S., Adams H., Spargo P. L. (1998). (1R,3R)-2-methylene-1,3-dithiolane
1,3-dioxide: a highly reactive and highly selective chiral ketene
equivalent in cycloaddition reactions with a broad range of dienes. J. Chem. Soc. -Perkin Trans..

[44] Payeras A. M. I., Ho R. Y. N., Fujita M., Que L. (2004). The reaction of FeII­(tpa)
with H_2_O_2_ in acetonitrile and acetone-distinct
intermediates and yet similar catalysis. Chem.-Eur.
J..

[45] Kaizer J., Klinker E. J., Oh N. Y., Rohde J. U., Song W. J., Stubna A., Kim J., Münck E., Nam W., Que L. (2004). Nonheme Fe^IV^O complexes that can oxidize
the C-H bonds of cyclohexane at room temperature. J. Am. Chem. Soc..

[46] te
Velde G., Bickelhaupt F. M., Baerends E. J., Fonseca
Guerra C., van Gisbergen S. J. A., Snijders J. G., Ziegler T. (2001). Chemistry
with ADF. J. Comput. Chem..

[47] Becke A. D. (1988). Density-functional
exchange-energy approximation with correct asymptotic behavior. Phys. Rev. A.

[48] Stephens P. J., Devlin F. J., Chabalowski C. F., Frisch M. J. (1994). ab-initio calculation
of vibrational absorption and circular-dichroism spectra using density-functional
force-fields. J. Phys. Chem..

[49] Perdew J. P. (1986). Density-functional
approximation for the correlation-energy of the inhomogeneous electron-gas. Phys. Rev. B.

[50] Grimme S., Antony J., Ehrlich S., Krieg H. (2010). A consistent
and accurate
ab initio parametrization of density functional dispersion correction
(DFT-D) for the 94 elements H-Pu. J. Chem. Phys..

[51] Swart M. (2013). A new family
of hybrid density functionals. Chem. Phys. Lett..

