# Supplementary material for: para-Donor Effects in PyNO Push Ligands Control O–O Bond Cleavage of TPAFe(III)-Acylperoxo to High-Valent Fe(IV)=O or Fe(IV)=O Radical Cation Species
Source: Inorg Chem. 2026 Feb 28;65(10):5560–8. doi: 10.1021/acs.inorgchem.5c05784 (PMC12997162; doi:10.1021/acs.inorgchem.5c05784)
Supplement: Supplementary file 1 [file ic5c05784_si_001.pdf]

Supporting Information

para-Donor Effects in PyNO Push Ligands Control O–O Bond Cleavage of TPAFe(III)-Acylperoxo to High-Valent Fe(IV)=O or Fe(IV)=O Radical-Cation Species

Chang-Quan Wu, Po-Chun Yang, Tzu-Hsien Tseng, Wan-Qin Zeng, Hui-Ling Cheng, Tao-Hsien Liu, Chung-Wei Li, I-Chung Lu*, and Peter Ping-Yu Chen*

Department of Chemistry, National Chung Hsing University, Taichung City 402, Taiwan (R.O.C.)

*Email: iclu@nchu.edu.tw; pychen@dragon.nchu.edu.tw

TD-DFT-Assigned Electronic Transitions

TD-DFT calculations of the absorption spectrum of compound **4** at the PBE0/TZ2P level of theory, employing the COSMO/MeCN solvent model, yielded a simulated spectrum exhibiting two prominent absorption peaks at 525 nm and 585 nm, attributed to α−spin manifold excitations (Figure S10a). These calculated absorption bands closely resemble the experimentally obtained absorptions at 586 nm and 637 nm. The absorption peak at 525 nm is primarily associated with transitions: 28% from Fe_d_{yz}-O_p_y (HOMO-4) to Fe_d_{x2-y2} (LUMO+1), 16% from Fe_d_{yz}-O_p_y to the unoccupied NMe_2-PyNO_π (LUMO), and 11.1% from Fe_d_{xy} to LUMO. In contrast, the absorption peak at 585 nm mainly comprises transitions, with 54.2% from Fe_d_{yz}-O_p_y (HOMO-4) to LUMO and 17% from Fe_d_{yz}-O_p_y (HOMO-4) to Fe_d_{x2-y2}. TD-DFT calculations were also performed at the B3LYP/TZ2P level of theory, affording two major absorptions at 567 nm (75% Fe_d_{xy}→ LUMO) and 661 nm (77% from (Fe_d_{xz}-O_p_x) → LUMO (Figure S10b). Thus, based on these two computed absorption spectra, the two peaks at 586 and 637 nm highly correlate with the NMe_2-PyNO_π orbital. TD-DFT attributes the 586 and 637 nm absorptions primarily to Fe/O→PyNO–NMe_2 π* metal-to-ligand charge-transfer (MLCT) transitions, with appreciable ligand-field (d–d) admixture involving the Fe_d_{x2-y2} orbital.

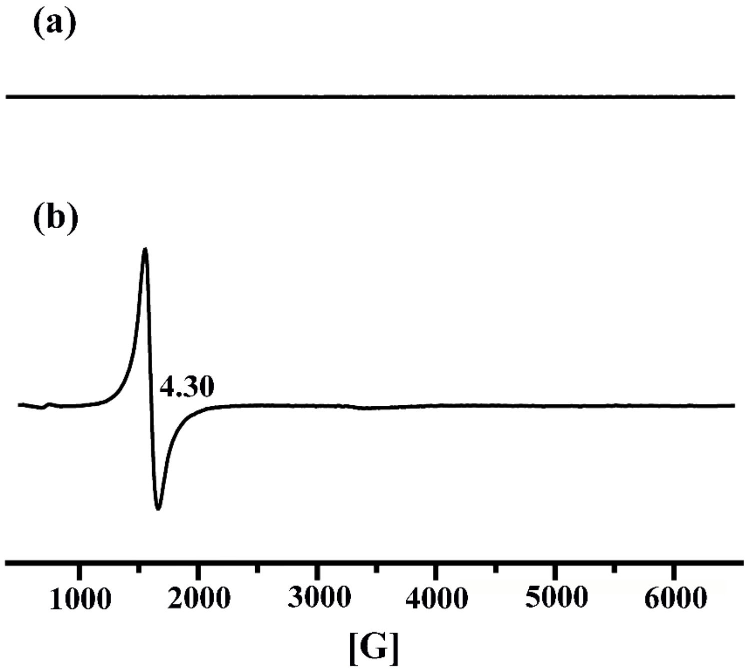

Figure S1. X-band EPR spectra (77 K) of MeCN solutions: (a) $[(TPA)(H_2O)Fe^{III}(\mu\text{-}O)Fe^{III}(H_2O)(TPA)]^{4+}$ (**1**); (b) after the addition of two equiv of Py-*N*-oxide (PyNO) at RT followed by rapid freezing. Acquisition: $\nu \approx 9.50$ GHz, power 0.2 mW, modulation 100 kHz, 20 G; center field ~3350 G.

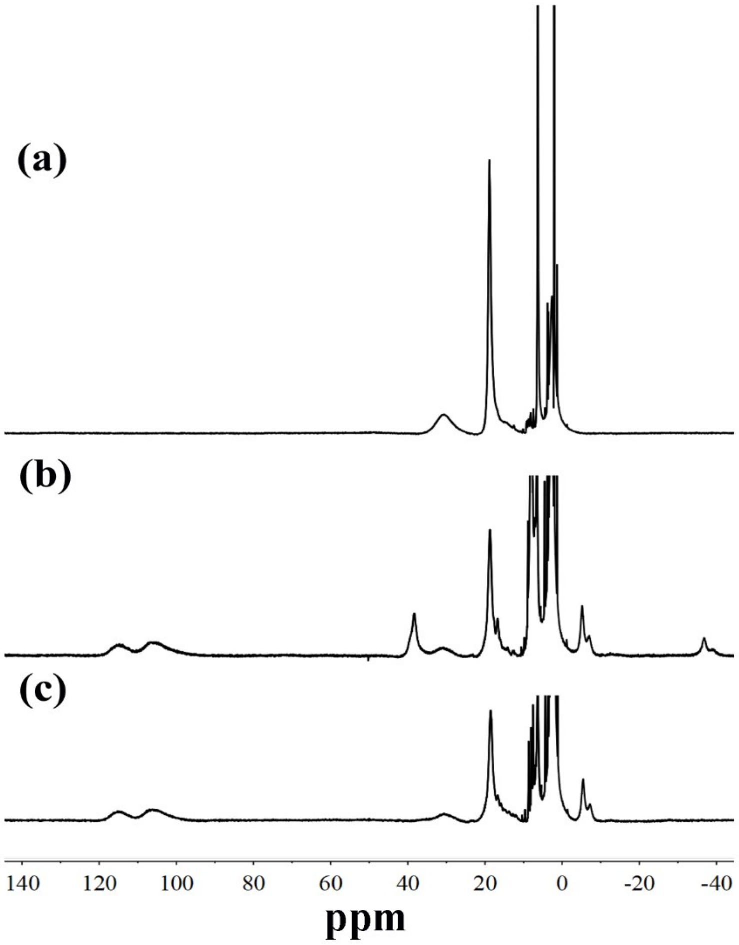

Figure S2. ^{1}H NMR spectra (600 MHz, CD_3CN, RT) of (a) $[(TPA)(H_2O)Fe^{III}(\mu\text{-}O)Fe^{III}(H_2O)(TPA)]^{4+}$ (**1**), (b) after addition of two equiv of PyNO, and (c) the addition of two equiv of PyNO-d_5 into the MeCN solution of **1** at room temperature.

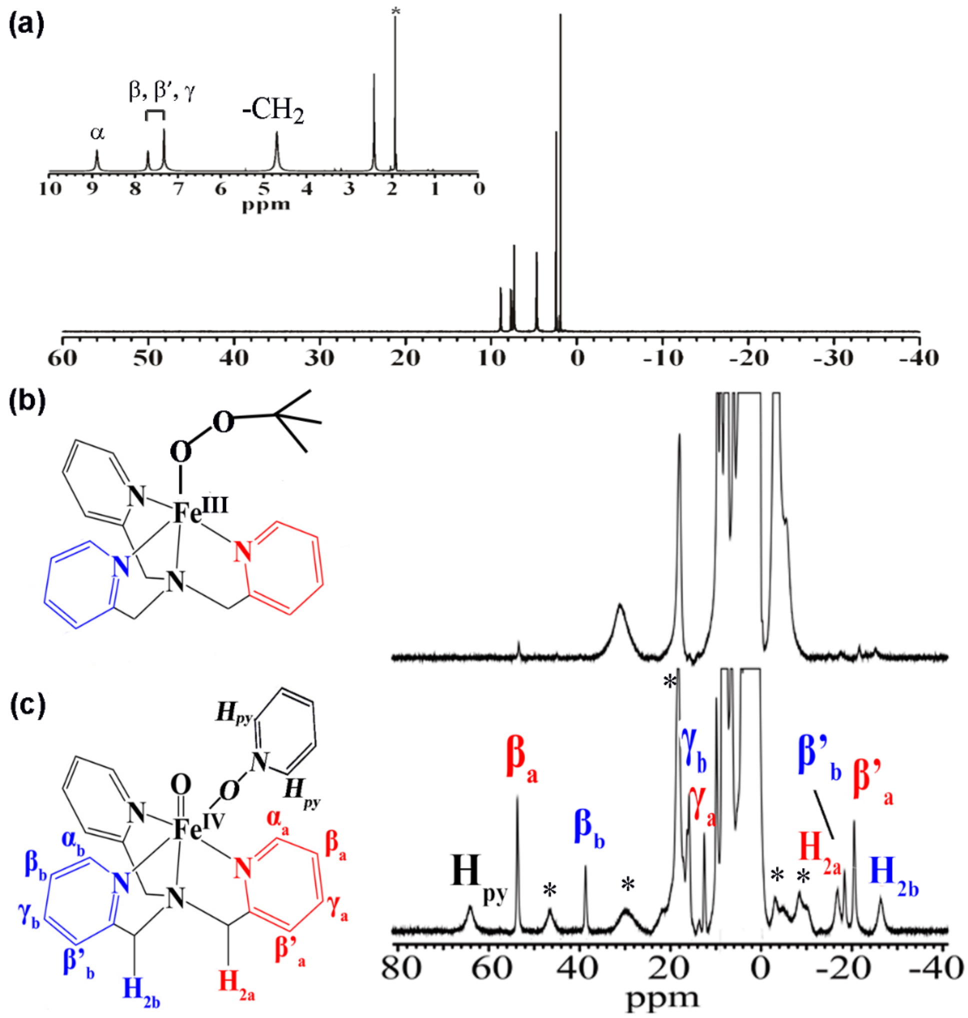

Figure S3. ^{1}H NMR spectra of (a) $[(TPA)(H_2O)Fe^{III}(\mu\text{-}O)Fe^{III}(H_2O)(TPA)]^{4+}$ (**1**) in CD_3CN at -40 °C; (b) after addition of 1.5 equiv of tert-butyl hydroperoxide (*t*BuOOH) to the solution of **1**; (c) after subsequent addition of 1 equiv of pyridine *N*-oxide (PyNO) to the mixture in (b) after 5 min. Peaks marked with * correspond to unidentified species.

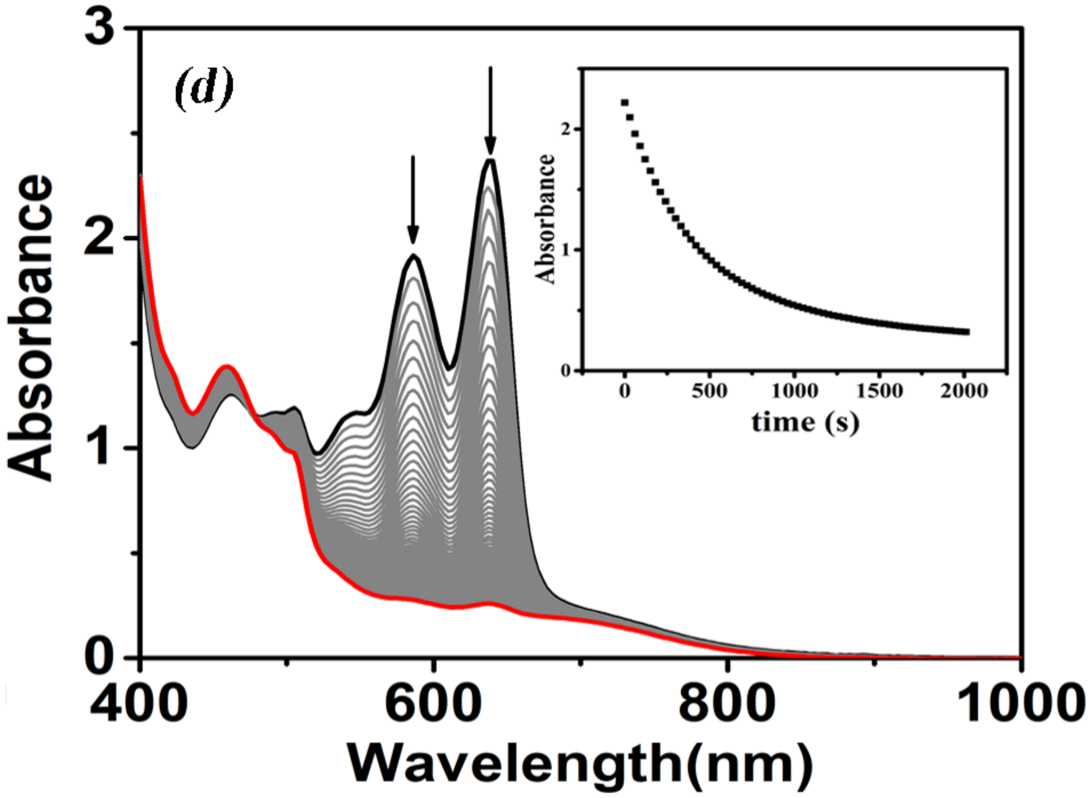

Figure S4. Decay of **4** at −40 °C monitored by UV–vis at 586 and 637 nm, evidenced by the decrease in absorbance at 586 nm and 637 nm at -40 °C. Inset: Time-dependent decay at 637 nm, fitted to a first-order kinetic model, yielding k_{obs} = 1.71×10^{-3} s^{-1}.

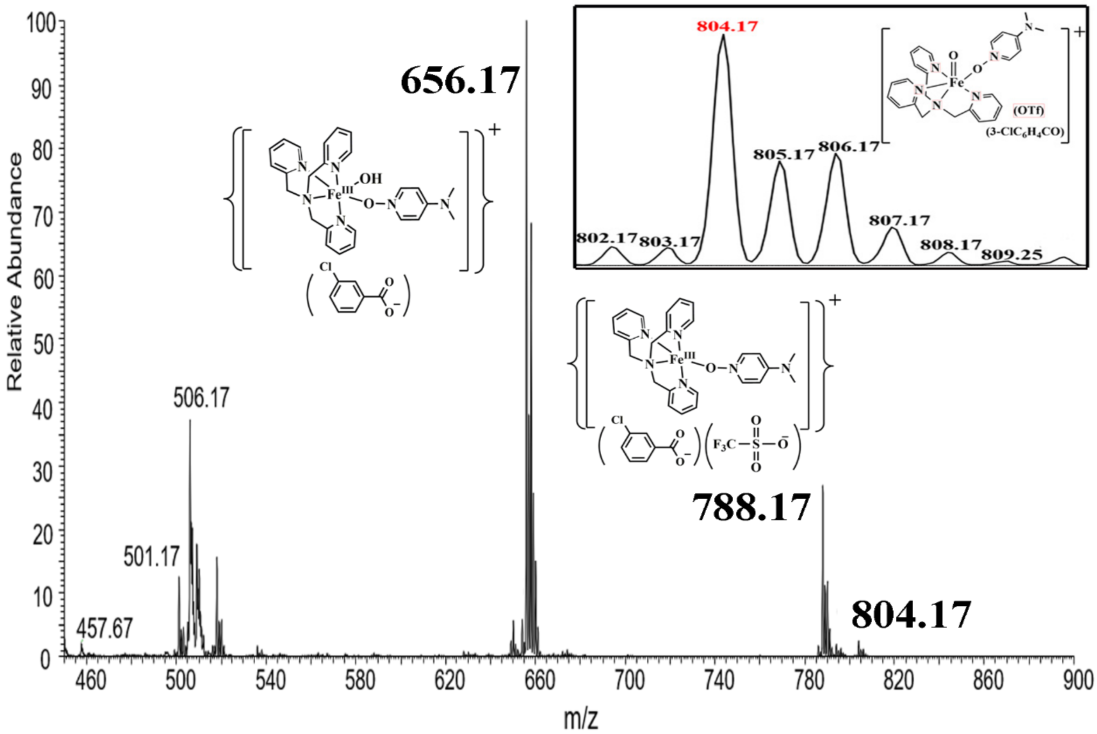

Figure S5. Cold-spray ionization mass spectrometry (CSI-MS) spectrum of the reaction mixture containing $[(TPA)(H_2O)Fe^{III}(\mu\text{-}O)Fe^{III}(H_2O)(TPA)]^{4+}$ (**1**) with two equivalents of PyNO and two equivalents of mCPBA in acetonitrile at -40 °C.

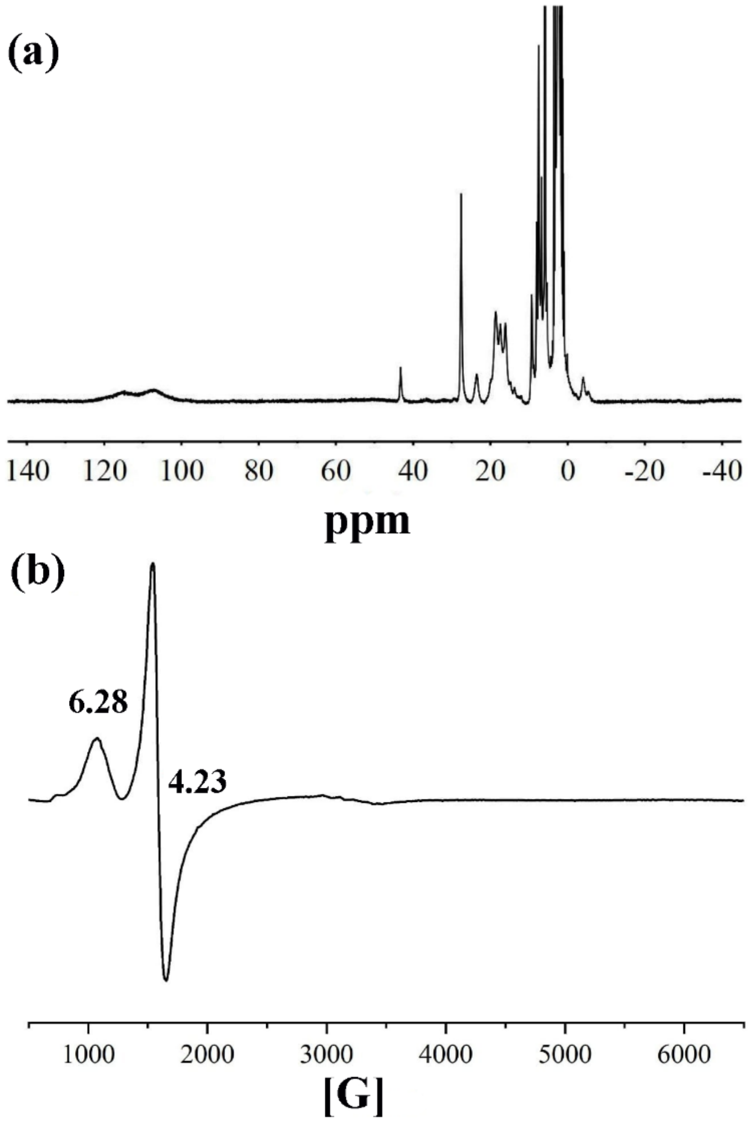

Figure S6. (a) Room-temperature ^{1}H NMR spectrum and (b) 77 K EPR spectrum recorded after addition of 2 equiv of NMe_2-PyNO to a CD_3CN solution of [(TPA)(H_2O)Fe^{III}(μ-O)Fe^{III}(H_2O)(TPA)]$^{4+}$(**1**).

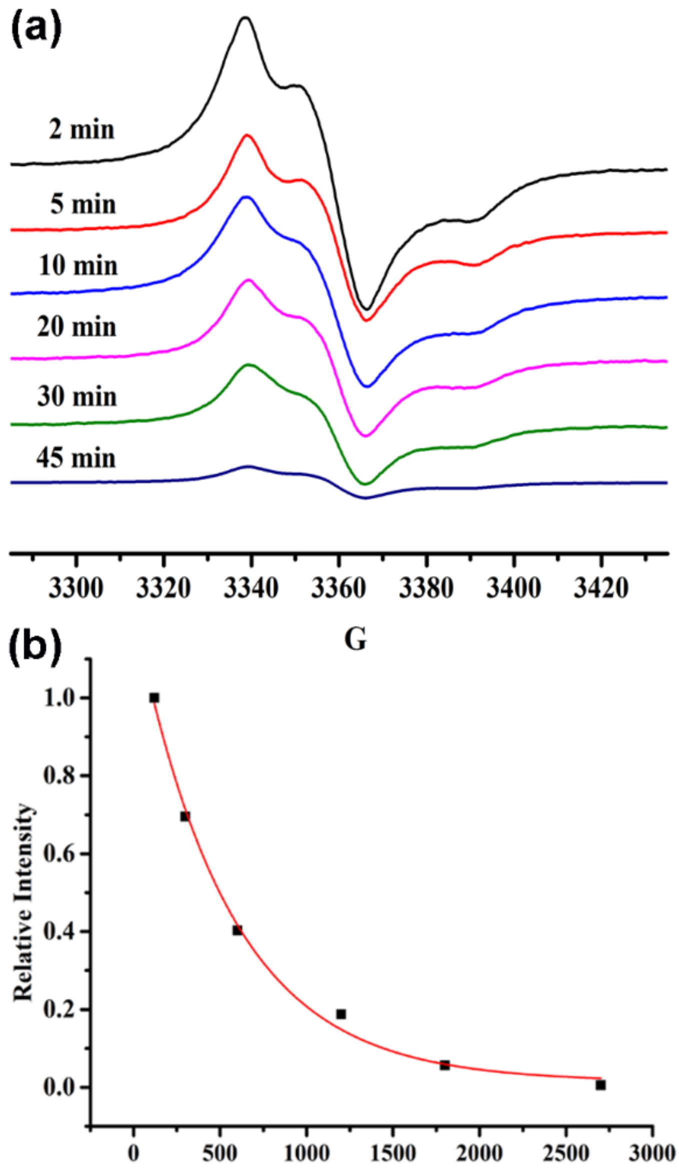

Figure S7. (a) Time-course decay of the EPR signal of $[(TPA)(NMe_2\text{-}PyNO)Fe(O)]^{3+}$ (**4**) assigned to a high-valent iron–oxo species, monitored at g = 2.024, 2.011, and 1.992 at −40 °C. (b) Time-dependent EPR intensity with a first-order fit, giving $k_{obs} = 1.84 \times 10^{-3}\ s^{-1}$.

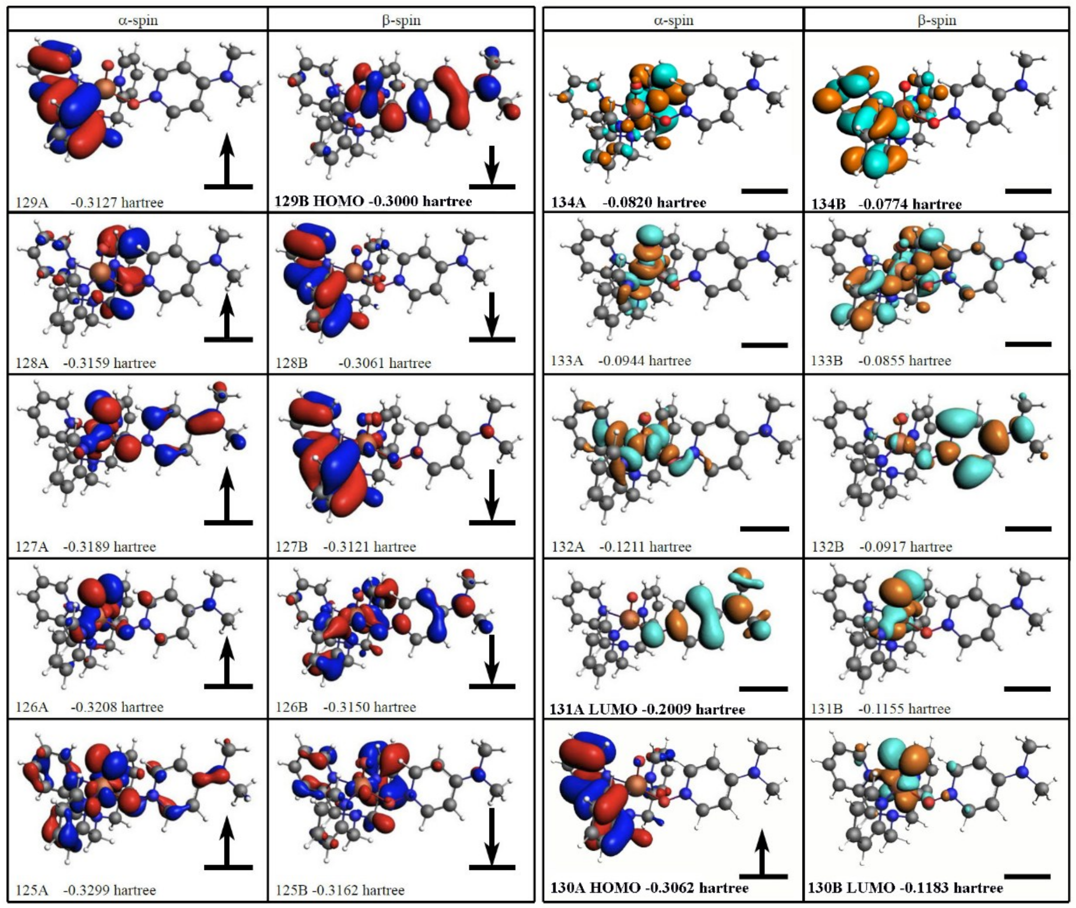

Figure S8. Ten key molecular orbitals (MOs) of complex **4** (S = 1/2), obtained from unrestricted DFT calculations at the COSMO (MeCN) PBE0/TZ2P level of theory. α-Spin (left) and β-spin (right) orbital sets are displayed side by side. All isosurfaces are plotted at an isovalue of 0.03 a.u.

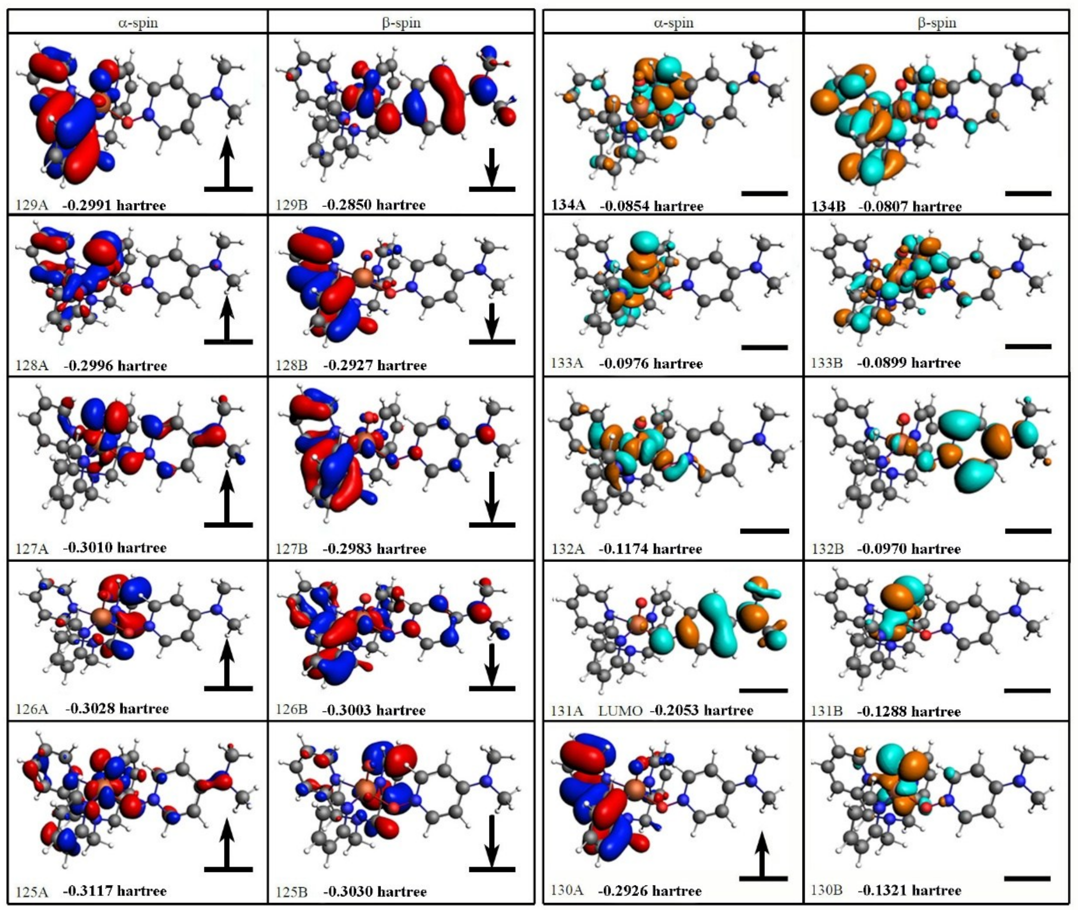

Figure S9. Ten key molecular orbitals (MOs) of complex **4** (S = 1/2), obtained from unrestricted DFT calculations at the COSMO (MeCN) B3LYP/TZ2P level of theory. α-Spin (left) and β-spin (right) orbital sets are displayed side by side. All isosurfaces are plotted at an isovalue of 0.03 a.u.

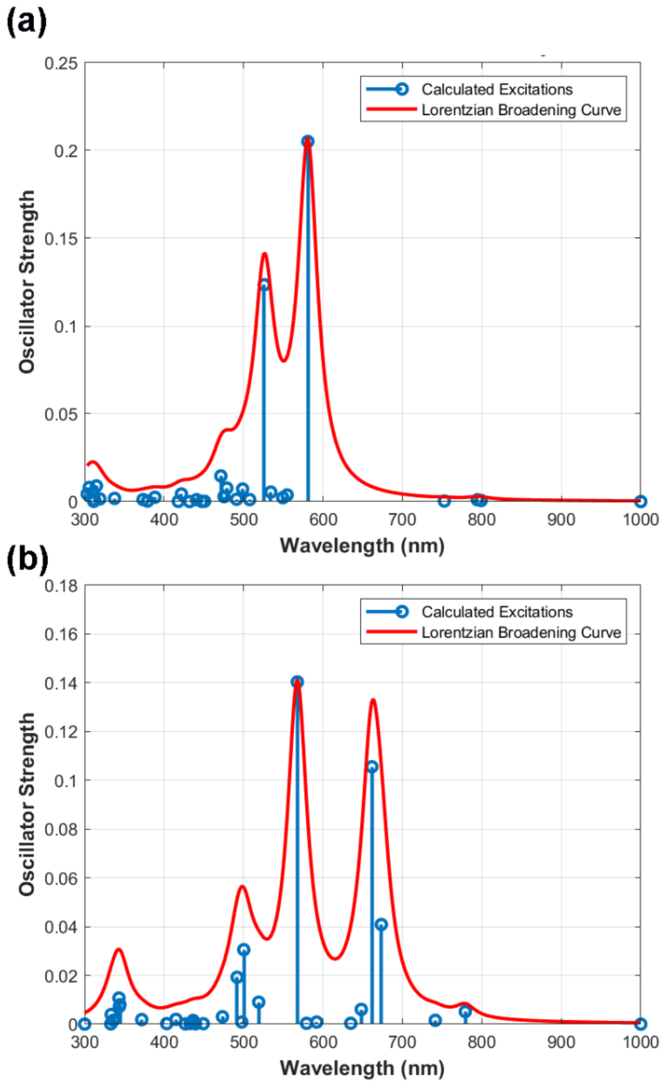

Figure S10. TD-DFT absorption spectra of the geometry-optimized S = 1/2 complex $[(TPA)(NMe_2\text{-}PyNO)Fe(O)]^{3+}$ (**4**) computed at (a) scalar-ZORA PBE0/TZ2P (COSMO/MeCN) and (b) scalar-ZORA B3LYP/TZ2P (COSMO/MeCN). Fifty vertical excited states were included.

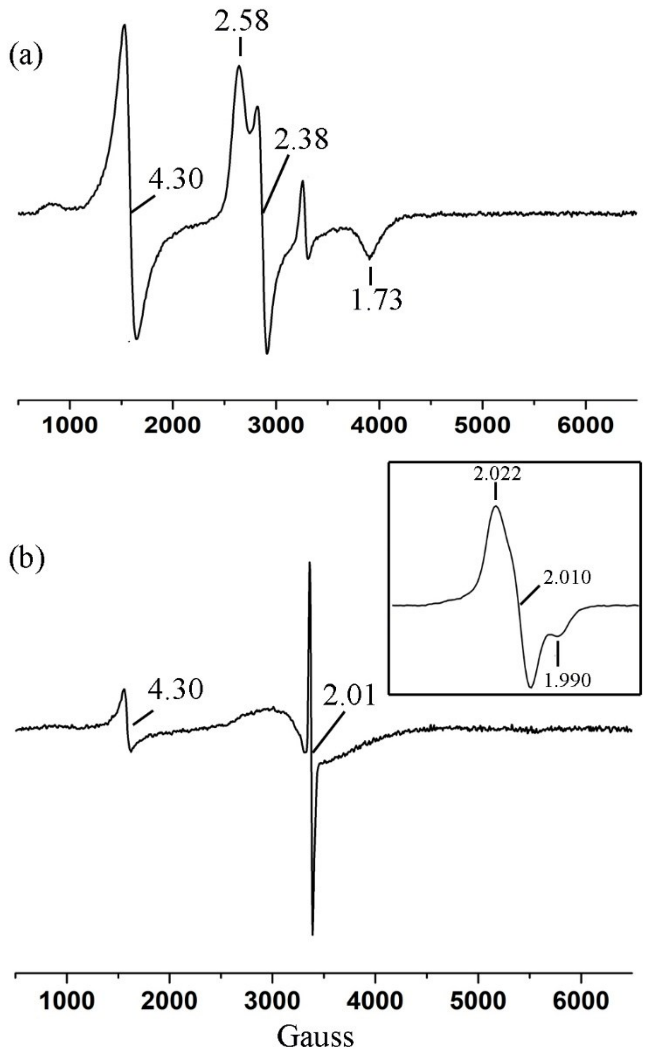

Figure S11. X-band EPR spectra (77 K) of frozen MeCN solutions of (a) $[(TPA^{*})Fe(II)(CH_3CN)_2]^{2+}$ after reaction with 5 equiv mCPBA at −40 °C, and (b) $[(TPA^{*})Fe(II)(CH_3CN)_2]^{2+}$ after addition of 5 equiv mCPBA and 3 equiv NMe_2-PyNO at −40 °C. Inset: expanded region collected with a 500 G scan window centered at 3410 G. Acquisition conditions: T = 77 K; microwave power = 0.2 mW; modulation amplitude = 20 G.

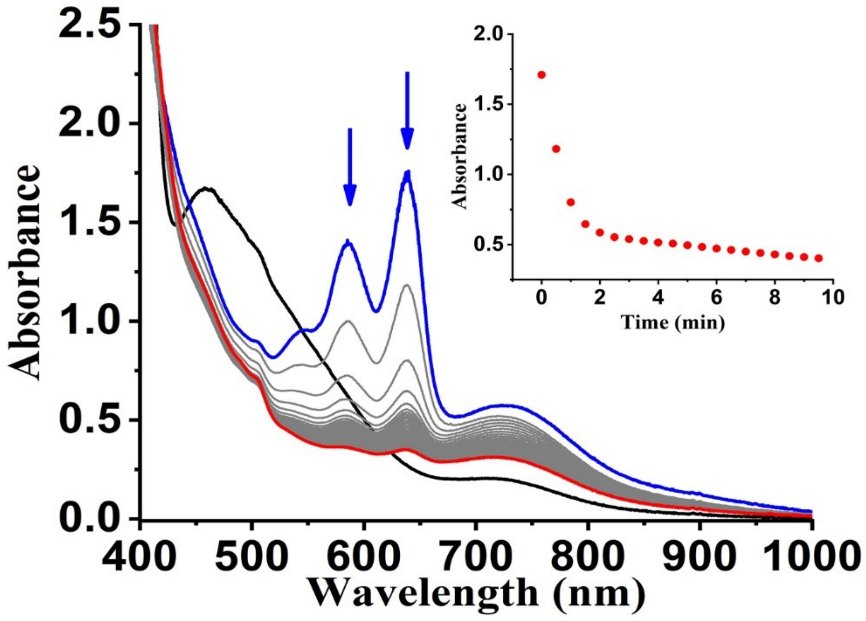

Figure S12. UV-vis spectra at −40 °C showing evolution from the precursor $[(TPA^*)Fe^{III}–acylperoxo]^{2+}$ (black) to the high-valent oxoiron species **4** (blue) upon addition of 2 equiv NMe_2-PyNO, followed by decay to the final product (red). Inset: kinetic trace at 639 nm with a first-order fit, yielding $k_{obs} = 1.72 \times 10^{-2}$ s^{-1}.

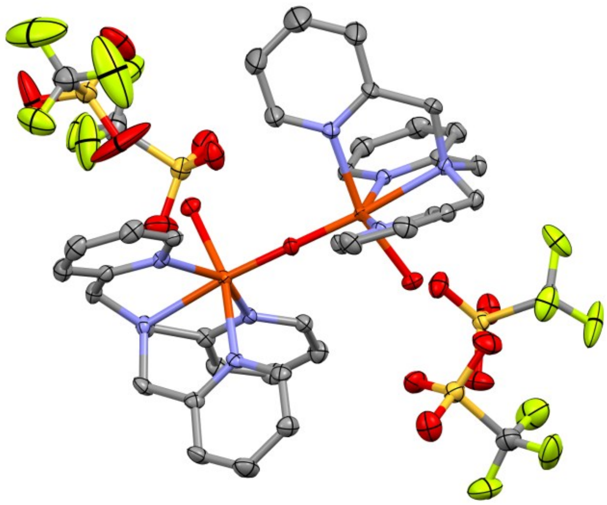

Figure S13. ORTEP diagram of [(TPA)(H_2O)Fe^{III}(μ-O)Fe^{III}(H_2O)(TPA)](SO_3CF_3)$_4$ (**1**) with 30% probability ellipsoids. Hydrogen atoms are omitted for clarity.

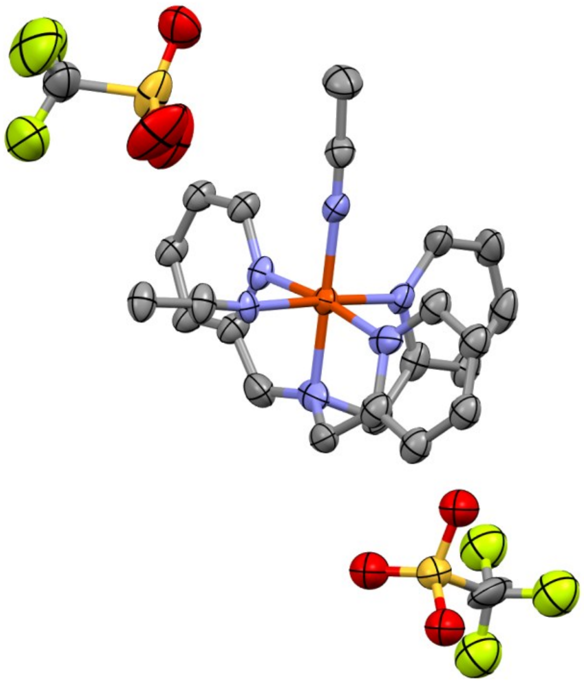

Figure S14. ORTEP diagram of [Fe^{II}(TPA)($NCCH_3$)$_2$](SO_3CF_3)$_2$ with 30% probability ellipsoids. Hydrogen atoms are omitted for clarity.

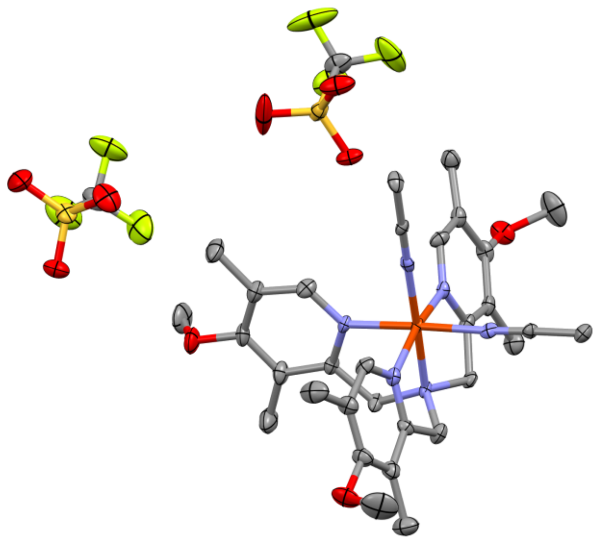

Figure S15. ORTEP diagram of $[Fe^{II}(TPA^*)(NCCH_3)_2](SO_3CF_3)_2$ with 30% probability ellipsoids. Hydrogen atoms are omitted for clarity.

Table S1. Literature DFT Fe=O bond lengths and Fe/O/Ligand spin densities for nonheme iron–oxo species, with compound **4** included for direct comparison.

Ironoxo species	Spin state	Fe=O (Å)	Spin on			Ref.
			Fe	O	Ligand	
$[(TAML)Fe^{V}O]^{-a}$	1/2	1.60	~0.64	0.22	Not reported	9,13
$[(TAML)Fe^{V}O]^{-b}$	1/2	1.64	0.69	0.32	0.02 (TAML)	30
$[(TAML^{\bullet+})Fe^{IV}O]^{-c}$	1/2	1.63–1.65	1.34	0.57	-0.39 (TAML)	30
$[(TPA)Fe^{IV}O(CH_3CN)]^{2+d}$	1	1.63	1.36	0.73	-0.11 (TPA)	31
$[(Me_3NTB)Fe^{IV}O(CH_3CN)]^{2+}$	1	1.60	1.18	0.87	-0.04 (Me_3NTB)	31
$[(TQA)Fe^{IV}O(CH_3CN)]^{2+}$	2	1.62	3.10	0.69	0.10 (TQA)	31
$[(TMC)Fe^{IV}O]^{2+e}$	1	1.65	1.36	0.81	Not reported	32
$[(N4PY)Fe^{IV}O]^{2+f}$	1	1.65	1.06	0.98	-0.04 (N4PY)	33
$[(TMP^{\bullet+})Fe^{IV}(O)(Cl)]^{g}$	1/2	1.65	1.35	0.82	-1.10 (por)	34
$[(TMP^{\bullet+})Fe^{IV}(O)(Cl)]^{g}$	3/2	1.65	1.12	0.87	0.98 (por)	34
$[(TPA)(NMe_2\text{-}PyNO^{\bullet+})Fe(IV){=}O]^{3+}$(**4**)	1/2	1.64	1.33	0.76	-0.88 (NMe_2-PyNO)<br>-0.10 (TPA)	This work

[a]B3LYP/6-311G; [b]BP/LACVP; [c]B3LYP/LACVP; [d] B3LYP/Def2-TZVPP; [e]PBE0/TZP; [f]UB3LYP/LACVP;[g] B3LYP/6-311+G*, LACV3P(For Fe)

Table S2. Summary of Fe–O bond distances (Mayer bond orders in brackets), Mulliken spin populations, and Mulliken charges (in parentheses) for compound **4** (S = 1/2) obtained from DFT calculations employing different functionals and basis sets.

**4**	Fe=O (Å)	Spin density on			
		Fe	O	Ligand	
				TPA	NMe_2-PyNO
BP86/TZ2P (gas)	1.65 [1.85]	1.10 (0.98)	0.81(-0.52)	-0.07	-0.74 (1.06)
BP86/TZ2P (CH_3CN)	1.64 [1.82]	1.13 (0.95)	0.73 (-0.53)	-0.06	-0.71 (1.12)
BP86-D3/TZ2P (gas)	1.64 [1.82]	1.05 (0.95)	0.76 (-0.50)	-0.09	-0.70 (1.03)
BP86-D3/TZ2P (CH_3CN)	1.64 [1.81]	1.10 (0.94)	0.71(-0.52)	-0.06	-0.66(1.09)
B3LYP/TZ2P (gas)	1.62 [1.98]	1.30 (1.11)	0.77 (-0.58)	-0.08	-0.90 (1.13)
B3LYP/TZ2P (CH_3CN)	1.63 [1.98]	1.29 (1.10)	0.77 (-0.61)	-0.07	-0.89 (1.21)
B3LYP/ET-pVQZ (gas)	1.62 [1.97]	1.33 (1.18)	0.76 (-0.82)	-0.09	-0.89 (0.92)
B3LYP/ET-pVQZ (CH_3CN)	1.63 [1.96]	1.32 (1.28)	0.75 (-0.86)	-0.07	-0.72 (0.98)

DFT_optimized xyz coordinates

62
C25H28FeN6O2 (**4**) optimized with BP86/TZ2P (gas)

Fe	4.79574187154607	17.88390224034937	7.90785433545627
O	5.46602952507765	16.98553074342218	6.70380921347884
N	4.03310288801965	18.96395945437447	9.53529323344388
N	3.34367430090380	16.63096802282570	8.42747616370040
N	6.02757475191214	19.44246236827365	7.82636130518057
N	5.94534070596384	17.06934896549597	9.26902394295510
C	2.57902028193002	18.60503283728266	9.61347306864107
C	2.41718922911778	17.14081996399555	9.28547962347105
C	1.37548608735961	16.35267492333822	9.76507501060826
C	1.27554606834567	15.02346882706369	9.34796833818533
C	2.22810505444877	14.51564081021261	8.46480994271749
C	3.25624035622030	15.34379718305931	8.02739599099059
C	4.28253048162058	20.40589662470550	9.20888546872537
C	5.63557930393741	20.52776512247909	8.55067910630657
C	6.43214273716428	21.66657207654981	8.62247587991987
C	7.64706925406378	21.69611315402471	7.93434917176794
C	8.03758968529261	20.57792583271099	7.19744887712635
C	7.20793168001318	19.46230228352375	7.16943721099928
C	4.76298945794989	18.56039067842929	10.79335070253353
C	5.79188370343989	17.49080673779101	10.54588424250666
C	6.56318131512433	16.95694588077983	11.57719896589331

C	7.50712767024112	15.97444654041195	11.28912674750022
C	7.65619141227346	15.54803800631503	9.96758454548770
C	6.86172620512911	16.11145542848313	8.98004415925147
H	2.15573493805266	18.85199107889145	10.59739447651961
H	2.04449026906765	19.20516698273705	8.86485131571273
H	0.64982759399789	16.77666409333672	10.45932186086132
H	0.46704679499296	14.39191744672239	9.71599914400684
H	2.18858598322441	13.48351011329812	8.11898628233943
H	4.03301932278839	14.99832627432188	7.34714711845989
H	4.20645429783428	21.04153087522371	10.10272545554944
H	3.50295863730389	20.72953093712100	8.50599192626005
H	6.10487710177981	22.52102901310077	9.21492144570835
H	8.28431974906536	22.57904849785167	7.98287975387660
H	8.98130823059204	20.55776619118591	6.65400039794019
H	7.46790477356812	18.55743211450396	6.62225280274039
H	5.24924048249715	19.44564925099735	11.22691309997974
H	4.03522406238096	18.21049107845534	11.53888046410220
H	6.41975485674097	17.31363251248515	12.59747412931082
H	8.11800424307921	15.54731583166188	12.08424468364794
H	8.38058901299099	14.78168432393163	9.69494436127912
H	6.93035256179600	15.81705756800926	7.93469677970706
O	3.46245941878428	18.85615677049274	6.78300782393253
N	3.29626570753424	18.97380624844578	5.46959626954257
C	2.28082040098784	19.81055423621615	5.05630044276633
C	2.00149244525888	19.98351694932702	3.73070876799577
C	2.75284114707351	19.28965332403808	2.72782457796494
C	3.79712864245400	18.43483426031807	3.20344935511058
C	4.05202646499356	18.29713966418478	4.54395043034198
H	1.72472693347213	20.29433035660060	5.85491616134026
H	1.17049076582602	20.63620023314185	3.47443016953506
H	4.44183766436452	17.89070813606338	2.51753805453302
H	4.83233050499961	17.66029025017872	4.96154268650247
N	2.48961346141510	19.43870144881498	1.40943459466398
C	1.55253757631366	20.45323541208208	0.90199622849367
C	3.12174441227446	18.59989745933162	0.38029304827292
H	1.54911936380154	21.34471551902615	1.53469537380935
H	0.53807874413676	20.02821365840982	0.83882353749907
H	1.86880471619347	20.73535347035171	-0.10815821576837
H	4.04819388690693	19.07796313562388	0.02444897569711
H	2.43147551501134	18.51983604481815	-0.46652294407591
H	3.33576736229115	17.59751783164442	0.75999064557105

62

C25H28FeN6O2 (**4**) optimized with BP86/TZ2P (CH_3CN).

Fe	4.59293808147815	17.56689835249761	7.88968740554132
O	5.21460226708598	16.50450397836926	6.80142859739655
N	3.92218786613494	18.85619774204937	9.40059655205305
N	3.24838996952581	16.37082654288343	8.70215221544933
N	5.73238495527221	19.12922834571225	7.46416544772174

N	5.88551522228020	17.01903848988771	9.26476022905315
C	2.50264473677743	18.46564677250808	9.66358005606430
C	2.37684672927080	16.97068532468903	9.55308609549986
C	1.40719370348978	16.22978519306694	10.21835517805687
C	1.32419516624187	14.85784903110796	9.98993395422485
C	2.21889037885346	14.25886735007032	9.10567551274747
C	3.17710794121495	15.04435285965044	8.47912764184772
C	4.06443692167524	20.23183625235302	8.83282615396929
C	5.34531470928922	20.29641162958264	8.04519773870362
C	6.08988109422051	21.45792773256081	7.87708240569368
C	7.24255207031428	21.42099701326304	7.09475334906726
C	7.63060468068976	20.21738792110107	6.50954431390141
C	6.85709613540473	19.08423324968855	6.72091942657845
C	4.77050320338015	18.69380182619346	10.62994209225765
C	5.81116104803507	17.62676630528866	10.46728133586739
C	6.67622208179856	17.28037730473614	11.50283515794868
C	7.63265012871474	16.29520646731590	11.29050750543323
C	7.70090834612754	15.67605874510300	10.04229300119007
C	6.81337201128268	16.05762660384777	9.04992114512553
H	2.16560457795743	18.83160303897182	10.64072843342047
H	1.87462086562815	18.93369113438347	8.89626239979791
H	0.72870484459334	16.73094272792298	10.90514632127456
H	0.57096958039297	14.26284860013388	10.50234503136976
H	2.18874638704506	13.19167542651809	8.90040550226259
H	3.90590974671553	14.63483038359799	7.78411208005048
H	4.03564659396100	20.99407581727642	9.62079870800722
H	3.21252666869037	20.41083926884322	8.16594232743296
H	5.76475744873095	22.37599072030937	8.36131062278265
H	7.83671998836548	22.32136501722110	6.95358403868357
H	8.52778473911297	20.14298106470842	5.90021300805052
H	7.11785525574375	18.11479451098710	6.30255948053205
H	5.26002450843352	19.64879693228915	10.85770440291904
H	4.12829205089480	18.46072448456571	11.48780379863298
H	6.58902739372840	17.78824408846478	12.46100842627452
H	8.31630189101480	16.01259030985495	12.08831884552165
H	8.43137899156825	14.89962952105365	9.82912388016988
H	6.81231402661826	15.60943173608633	8.05983681754207
O	3.13505844002576	18.26798026157683	6.81659929056901
N	3.16430960724447	18.67190575383525	5.55252529456767
C	2.16535427376861	19.53077189737578	5.16944600887892
C	2.07289408997242	19.95294819981348	3.87729930863517
C	3.01238468460507	19.50260143943936	2.90158376417968
C	4.03084306621702	18.60896747464430	3.34678680281632
C	4.08903361391785	18.21418776673331	4.65330891947621
H	1.47100748095838	19.81315853053583	5.95355379086249
H	1.25279789416857	20.61395644351155	3.61975683665155
H	4.79606237887701	18.23660714848479	2.67439347805965
H	4.82182857315770	17.51760613022981	5.05215555950119
N	2.94320892964202	19.90666602847954	1.61919526489345

C	2.01427227591293	20.94851932063481	1.18348552926167
C	3.79683326766583	19.33161000191426	0.58031636547425
H	1.87221708510190	21.69726734412845	1.96501836059207
H	1.04811641784986	20.49606314380072	0.91797881243846
H	2.43342105864906	21.42776274167343	0.29563425114664
H	4.75483650972659	19.86979955146438	0.54679673435062
H	3.29152029711242	19.45350543006605	-0.38048299424388
H	3.97292070163738	18.26983017890407	0.76420017424955

62

C25H28FeN6O2 (**4**) optimized with BP86-D3/TZ2P (gas)

Fe	4.57313188139694	17.66282243393975	7.85463538852944
O	5.18573774968903	16.65099745094962	6.72281498035255
N	3.90690688743369	18.90267096119715	9.41092837296464
N	3.24248309149644	16.44144248361530	8.64447520039540
N	5.76324966245403	19.21220925404826	7.52865692747849
N	5.84886494196339	17.04505165825565	9.21530142304816
C	2.47879834495192	18.50924460023956	9.63702005706031
C	2.37823351048246	17.00927450858017	9.52958515117449
C	1.45895079763186	16.23337948822466	10.22577529678101
C	1.42190350686592	14.85443341303021	10.00105610797297
C	2.30979536210298	14.28870759472136	9.08617025325058
C	3.21714117574899	15.11100234130112	8.42559236261789
C	4.08349333175220	20.29462625619237	8.89050789567829
C	5.40687109510780	20.35963567830849	8.17069844595251
C	6.22774958323302	21.48181190874595	8.13730249426236
C	7.43305506552870	21.42642230524724	7.43221939309054
C	7.78788148627718	20.24298809575897	6.78297443694562
C	6.93244528116408	19.14897697768403	6.85734012772437
C	4.74063531909896	18.67508640219589	10.64444626918676
C	5.77178267302205	17.60069703967684	10.44495483897686
C	6.62126053382813	17.19614831392359	11.47301699777232
C	7.56721681425523	16.20370646717937	11.22816762578923
C	7.63839895742245	15.63880472430886	9.95277877608973
C	6.76591115685714	16.07760482683607	8.96783891156569
H	2.10509345177229	18.88277000903535	10.60018545845418
H	1.87642688021039	18.96688924902715	8.84190446952344
H	0.78058922890764	16.70443562150932	10.93675081085130
H	0.71003952903501	14.23007247485351	10.54080306270818
H	2.31266508736452	13.21853002937354	8.88469545070921
H	3.94269405186914	14.73148150758625	7.70862382178609
H	4.01514246994266	21.04142240726668	9.69390272360546
H	3.26881221011183	20.48936245144920	8.18079989965335
H	5.93136550604473	22.38553792980625	8.66959344086738
H	8.09339503736727	22.29320070868915	7.40526666638116
H	8.72597658233943	20.15505904679095	6.23652205927099
H	7.16258297446072	18.18966338001450	6.39641976151283
H	5.24059634018511	19.61341680320498	10.92184918893967
H	4.08503601361976	18.40979305731022	11.48481962817505

H	6.53529135352236	17.66009891493944	12.45577280854007
H	8.23815531752215	15.87408268609238	12.02134417409538
H	8.36013722209059	14.85838761933510	9.71571365467237
H	6.76596392475993	15.66959340911441	7.95942425547119
O	3.14175867944044	18.43365634381385	6.78489354695239
N	3.15847758843670	18.79830981590002	5.50269576114212
C	2.13364073213559	19.62014164083372	5.09136705105343
C	2.00423925474730	19.98054669380470	3.78033863424541
C	2.92828026269526	19.50192041190146	2.79540743312969
C	3.98594460265445	18.66576153221682	3.27507822168626
C	4.08311626411473	18.33502321762109	4.60109271620400
H	1.43810966922740	19.91825249751968	5.87185345213445
H	1.15348080034322	20.60246152215262	3.51294010947062
H	4.75633868394304	18.28467697168464	2.60945446862190
H	4.84846092184225	17.68155998747179	5.01748882212487
N	2.80644598300575	19.82523436247823	1.48959164594540
C	1.85093437654309	20.84084541302395	1.01905076590620
C	3.61582241944614	19.17900339453617	0.44547393806976
H	1.73886040104481	21.64901294843981	1.74792189239732
H	0.87429644639280	20.37167400274244	0.82070787185323
H	2.22863292035123	21.26206370086644	0.08185383850559
H	4.55816871715469	19.73198227500315	0.30577093743216
H	3.05487131129272	19.21034858773252	-0.49423195404986
H	3.82470064723889	18.13461548954057	0.69497850587475

62

C25H28FeN6O2 (**4**) optimized with BP86-D3/TZ2P (CH_3CN)

Fe	4.49219509804062	17.32547123825157	7.82456297783251
O	5.14262538505047	16.18093781409967	6.85480572432790
N	3.80347879366231	18.78544715661940	9.16775191248471
N	3.25096379098837	16.19345777347345	8.83438045582097
N	5.53907549634933	18.86396399244975	7.18041892691660
N	5.81935588837111	17.01519470984324	9.23206706674340
C	2.42265160819063	18.35276358404444	9.54688131983898
C	2.39288434354552	16.85388696710222	9.65504197360141
C	1.51991399263568	16.15622707834889	10.48035483972227
C	1.52043352426117	14.76216502272369	10.44524033265177
C	2.40104780244151	14.10038453802026	9.58998107450630
C	3.26469184482099	14.84719776981684	8.79952916536947
C	3.83331705913863	20.06842986120911	8.40372719038475
C	5.08321590929228	20.08633743653611	7.56641496602012
C	5.74274852558025	21.24684656321800	7.18027153658839
C	6.88627968139128	21.14760247326771	6.38771398760555
C	7.35508789149779	19.88666870285170	6.01920672044543
C	6.66117077082122	18.75957782722969	6.43910115157910
C	4.71406031736104	18.86085384835832	10.35880405081529
C	5.73641614052356	17.76665980794290	10.35064735755313
C	6.59321445036428	17.55194777263271	11.42702262328320
C	7.55526113636383	16.55179127227018	11.34265261466304

C	7.63608226995924	15.78707584349884	10.17788350303438
C	6.75233082224849	16.03805426241499	9.14094223750373
H	2.09282427915795	18.83995241602637	10.47174002875501
H	1.74389970785339	18.65702381539255	8.74186884292498
H	0.85129883566128	16.70761202547843	11.13833451512113
H	0.84314078375073	14.19948323927048	11.08512390121678
H	2.43360794372658	13.01489627679080	9.53451503582431
H	3.98489012413088	14.39602836318651	8.12156630529639
H	3.77896000682117	20.93579703007759	9.07235634220937
H	2.94919157561009	20.09923725713125	7.75758056172225
H	5.36229809844315	22.21174120499536	7.50848596339021
H	7.41403442699565	22.04654285623885	6.07542814257274
H	8.25267461846292	19.76684992630660	5.41728127301625
H	6.97208972048067	17.74751782613044	6.19050682736812
H	5.23223652151040	19.82785516076202	10.34953270871303
H	4.12315417634853	18.82734731982125	11.28160883151690
H	6.49417230434595	18.17306151379113	12.31511913573640
H	8.23206039648555	16.36926547103057	12.17506300059847
H	8.37033094644425	14.99291483921842	10.06554437214830
H	6.75478977242271	15.46941652831085	8.21524679766330
O	2.97186567083301	17.81561713442503	6.74332635943545
N	3.06758880559051	18.40814352577664	5.55185792716580
C	2.13494050696239	19.36913296108902	5.26045552686373
C	2.15995706445073	20.01560450681244	4.05914488780952
C	3.15105386372210	19.68767399629740	3.08436836349800
C	4.08050192509165	18.65717780470708	3.42231696140324
C	4.02108093967930	18.04098959386834	4.63991850123934
H	1.39383823290951	19.54845108217127	6.03287313921870
H	1.39216643209792	20.75828461320371	3.86944773915732
H	4.86310622143021	18.34556107337702	2.73829849549372
H	4.68649420155460	17.24387499956836	4.95698893385069
N	3.20656588162228	20.32089916265522	1.89605707834536
C	2.35810421607137	21.47832244872904	1.60166822087775
C	4.11951872626995	19.87775816360434	0.83802033587284
H	2.25456823073262	22.11769021047385	2.48232486992792
H	1.36659167349779	21.13623266754999	1.27144066602007
H	2.82343220451867	22.05020829604596	0.79639564311288
H	5.14163291078233	20.21463875892922	1.06236156383920
H	3.79734613758498	20.32341041195276	-0.10442127450762
H	4.10193144598724	18.78747550142283	0.74722649486697

62

C25H28FeN6O2 (**4**) optimized with B3LYP/TZ2P (gas)

Fe	4.92515894028123	17.96311356160020	7.92615371620952
O	5.60488412993257	17.15839961539908	6.69331838447302
N	4.07900288494191	18.97458418176081	9.56822744660618
N	3.40725941550647	16.71263016302975	8.30297521368035
N	6.14393352263952	19.54408067722785	7.96327691831551
N	6.00382333192589	17.07549860584315	9.29266041877456

C	2.62459270690701	18.63326105556332	9.55168003438362
C	2.45758188579061	17.19098396124202	9.13933785130882
C	1.39210818008358	16.40128661708234	9.54018984696435
C	1.29638080555865	15.09784445041599	9.06320393033202
C	2.27564317749784	14.61794851231686	8.20304822761998
C	3.32279842559887	15.45148369530743	7.84760480723074
C	4.35736652485244	20.42468026525518	9.34017378693451
C	5.72965975905071	20.57852866321573	8.73037389947519
C	6.51207908274560	21.71014282837439	8.88910709726965
C	7.73975339775565	21.78293957413098	8.23841976314153
C	8.15178039502532	20.71740788128320	7.44806629165375
C	7.33079528602631	19.60805235641409	7.33734012200335
C	4.73997762920190	18.48366333452543	10.82778864473155
C	5.78771642585093	17.43311854070119	10.57049348691886
C	6.50603283260756	16.85455753459948	11.60758104925833
C	7.46349584249595	15.89337090986885	11.31900205603585
C	7.67862473538393	15.53295038028103	9.99308131272352
C	6.93343208074300	16.14119056886610	9.00237304256905
H	2.15617148626926	18.82998373473990	10.51730049480381
H	2.13798340507483	19.27345340102527	8.81579721161598
H	0.64843679382860	16.79841408589131	10.21843159707865
H	0.47323122219015	14.46495543327884	9.36849945762862
H	2.24131539072540	13.60822786130885	7.81814545888123
H	4.11848907432673	15.12517817277243	7.19308433199462
H	4.26197478640505	20.99940877074223	10.26268455244242
H	3.61260118543113	20.80374042663254	8.64040171171807
H	6.16894750863248	22.52222187529049	9.51621915064972
H	8.36601641310188	22.65800059119876	8.35297078692887
H	9.10038826474124	20.73438206688267	6.92977546514162
H	7.60668514961572	18.74647821512087	6.74608780009880
H	5.19528686324866	19.32805381146627	11.34669791414512
H	3.98080419499699	18.08186118625271	11.49987325188390
H	6.31457611731011	17.15777937241899	12.62865161080319
H	8.03315759741385	15.43264520659053	12.11541133277621
H	8.41519290331336	14.78922544551617	9.72248311143937
H	7.05852718361411	15.90125060405749	7.95766947429806
O	3.60035818024015	19.10010970881030	6.68070749177287
N	3.33386856057364	19.10456136658861	5.41006911246226
C	2.39159022984460	20.00670492895685	4.96748626503825
C	2.05220926637032	20.08694280710501	3.65629962294221
C	2.65542225696108	19.21872018675021	2.69001716739528
C	3.62556634486216	18.29581020332787	3.19481661261924
C	3.94792311658806	18.25957526065923	4.51732298152998
H	1.95538834018538	20.62530231327465	5.73654347845703
H	1.29300019227275	20.80198419835282	3.37966806434971
H	4.15436501608047	17.62209425938283	2.53846879292891
H	4.68650181234854	17.59858564131366	4.94948292346250
N	2.32771609229198	19.26985501521125	1.39184604291804
C	1.46051531731639	20.32122584471081	0.82985678075440

C	2.79932578930488	18.27720532402681	0.40996465377223
H	1.57432869871261	21.25661714472946	1.36737591599963
H	0.41934336513804	19.98851680130143	0.85870947819576
H	1.74663442704312	20.47277333433430	-0.20822769927101
H	3.72408993819098	18.63273876026673	-0.05203285448711
H	2.04015423863148	18.18414908635995	-0.36341101402471
H	2.95447395134094	17.30818291788918	0.87175084881979

62

C25H28FeN6O2 (**4**) optimized with B3LYP/TZ2P (CH_3CN)

Fe	4.59125223776185	17.54787410786189	7.88264845568241
O	5.17731505351623	16.50429154904474	6.77914978786461
N	3.91874777070499	18.84996688079962	9.39584137543093
N	3.21649092844196	16.36688894564345	8.70008870742380
N	5.74109170770398	19.13211286605183	7.46194482299185
N	5.88746586051159	16.99599486540738	9.26198434624338
C	2.49911287814835	18.47135653646853	9.65397702458261
C	2.35967500672270	16.97602830852251	9.54724206402483
C	1.39213850545758	16.25315137975316	10.22450448175861
C	1.29933261663303	14.88415443872479	10.01088056078511
C	2.17989918882192	14.27225245556797	9.12879265529854
C	3.13317037268719	15.04538024459165	8.49035231621789
C	4.07615005214505	20.22382752160922	8.83772972774660
C	5.36182519767891	20.28644437378965	8.05532591308215
C	6.11205231424621	21.44150557587555	7.91466501109503
C	7.26652837051685	21.40896655681869	7.14266536748351
C	7.64668590471773	20.21789908583126	6.53892599672001
C	6.86261541647471	19.09372038265190	6.72523258145051
C	4.76236500123884	18.66843002710280	10.62238475063752
C	5.82349597550576	17.62091569026060	10.44785851405745
C	6.70983054485280	17.31413962709037	11.47134733735578
C	7.67694014823934	16.34589373159742	11.26095162809807
C	7.73257446388827	15.70347176595318	10.02908795752487
C	6.82363537017007	16.05039552065923	9.05077919023711
H	2.16362532323999	18.83659462657538	10.62334305592541
H	1.87960121175437	18.93698162044043	8.88996610826056
H	0.72651749288420	16.76030791198426	10.90771175947334
H	0.55156521169912	14.30289107389757	10.53157805934677
H	2.14161761294296	13.21033634728174	8.93733204908880
H	3.84834031638927	14.62495752995430	7.79971838050323
H	4.05309848827239	20.97711147589741	9.62401557233982
H	3.23800276678493	20.41648152251907	8.17106573475171
H	5.79556831817691	22.34825735306973	8.40937269922282
H	7.86564935034974	22.30060604175904	7.02382624524823
H	8.54134216882460	20.14873844798063	5.93839599557630
H	7.11840034092883	18.13849368138896	6.29145711150219
H	5.23292116484168	19.61669744388700	10.87906056769718
H	4.12457216163022	18.40258393416310	11.46393553816200
H	6.63441752728049	17.83665174007432	12.41419905721313

H	8.37761416047325	16.09572782222460	12.04476982529953
H	8.46974275519584	14.94260236103207	9.82090538852725
H	6.81953306729894	15.58796468977435	8.07674809092924
O	3.10044555459179	18.28591373781829	6.75910821442093
N	3.10019831992242	18.65262532771086	5.50474802713822
C	2.09178537705049	19.49328936181348	5.11567651886492
C	2.00666408616659	19.92726397712682	3.83352326384951
C	2.96140960527994	19.49987410376563	2.86424865581910
C	3.97499386223237	18.60189517935391	3.30811960803225
C	4.02692221191616	18.20272983507463	4.60769524733106
H	1.39683268809019	19.76585026125939	5.89203695022965
H	1.19029958329948	20.57990612264978	3.57662442434606
H	4.73696840720428	18.23474724989572	2.64174790746158
H	4.75909726194852	17.51760332626856	5.00228616269176
N	2.90916100081628	19.92586122220324	1.59657268832622
C	1.97274251453908	20.96459241460183	1.15907161910369
C	3.78984538191151	19.39161452335308	0.55434879767046
H	1.79808546718445	21.68577962708865	1.94829937689912
H	1.03312433334181	20.49723502495121	0.85930517611914
H	2.40783334117723	21.46629901792413	0.30120051386401
H	4.72413981132757	19.95579287411439	0.54964548401852
H	3.29391261504847	19.52144608389032	-0.40155412790320
H	3.98946032413836	18.33926596639735	0.71791040743353

62
C25H28FeN6O2 (**4**) optimized with B3LYP/ET-pVQZ (gas)

Fe	4.91902905512242	17.95798615341682	7.92528017755680
O	5.59607808108558	17.14862673961165	6.69290265126773
N	4.07891804826183	18.97624293979879	9.56388994838650
N	3.40612982187396	16.70612184452367	8.31028591965934
N	6.13327983942675	19.54019711432786	7.94804044728025
N	6.00036571100641	17.07339291136175	9.29130379393430
C	2.62807843864144	18.62442927358150	9.56376301902005
C	2.46565107171401	17.18049493943756	9.15762572772604
C	1.41020580388493	16.38642825177474	9.57330550925365
C	1.31407006585263	15.08300061755970	9.09810631599787
C	2.28307672409538	14.60801095644035	8.22382697881600
C	3.32111375440007	15.44579054534038	7.85538853124965
C	4.34596353330709	20.42477849270555	9.31988640202590
C	5.71520760520257	20.57944742829816	8.70508972311530
C	6.49245222849027	21.71541209773405	8.85227645531172
C	7.71847744554068	21.78740015485628	8.19967675911565
C	8.13513782523364	20.71571426175309	7.42094080477972
C	7.31878831970221	19.60285402992769	7.32106964245818
C	4.75590369879274	18.50334029178298	10.82054446170890
C	5.78480016561789	17.43393685773590	10.56728713190570
C	6.49209279615380	16.84731916971191	11.60645019156662
C	7.43875835835551	15.87549068370576	11.32144617918796
C	7.65566385781568	15.51433384329296	9.99624326415104

C	6.92021943450776	16.12997096405526	9.00385171077083
H	2.16841624697106	18.82128058490045	10.53195329835062
H	2.12951557804226	19.25776568696619	8.83208405795193
H	0.67537219675257	16.77958984513140	10.26070088550322
H	0.49938635949343	14.44723590619314	9.41372245009899
H	2.24748168664975	13.60024827633267	7.83877384635463
H	4.10881799411461	15.12435017192287	7.19160337131304
H	4.24951947620233	21.00857975567121	10.23507176521349
H	3.59765798455553	20.79055467149387	8.61930632070748
H	6.14682717770501	22.53056224017610	9.47102297401868
H	8.34060649717524	22.66435335288346	8.30600191840055
H	9.08264499310662	20.73122591800408	6.90421789747594
H	7.59670509357957	18.73753440535366	6.73924514440318
H	5.23353173352649	19.35131298969282	11.30971272264262
H	4.00595872907805	18.12981223529055	11.51640514846120
H	6.30024855550905	17.15158105092255	12.62534274855681
H	7.99807694210844	15.40780311660812	12.11875731192085
H	8.38378734447864	14.76387071853045	9.72823562680388
H	7.04423931098404	15.88830563388297	7.96111011483326
O	3.59110680599464	19.08773220163524	6.68735887870707
N	3.33141937876816	19.09972729079895	5.41747098152063
C	2.39159244384836	20.00450423806106	4.97529484985006
C	2.05789083890021	20.09073529300094	3.66336368782776
C	2.66615049224783	19.22865135907642	2.69558084015262
C	3.63516235620123	18.30514574910213	3.19920136851232
C	3.94980737331806	18.26060816739468	4.52302197702821
H	1.95232269285957	20.61743570835411	5.74456112803337
H	1.30150870766621	20.80630885849066	3.38715755682608
H	4.16682340642708	17.63649670363653	2.54281746283519
H	4.68573847242160	17.59829375546422	4.95357872943987
N	2.34373822015210	19.28536877717391	1.39693493123885
C	1.47173722071792	20.33273193971245	0.83724202755390
C	2.82793613077830	18.30399088309406	0.41128464727661
H	1.56873078499757	21.26296377509103	1.38405241472194
H	0.43520710329786	19.98932744974730	0.85020963322044
H	1.76693319421167	20.49880504757241	-0.19470285062523
H	3.75451628931584	18.66620899996738	-0.03899232880887
H	2.07761687897202	18.21470400826868	-0.36915746399155
H	2.98224769772645	17.33220597050968	0.86434890800190

62

C25H28FeN6O2 (**4**) optimized with B3LYP/ET-pVQZ (CH_3CN)

Fe	4.55762762997772	17.51098911044682	7.88327534330888
O	5.13725062120579	16.45244689864763	6.79019355860918
N	3.89320294589864	18.83191564774658	9.38097045158218
N	3.20177122598706	16.33474081258188	8.73385004881757
N	5.69207342477933	19.09666548557882	7.42226309488399
N	5.87745061249871	16.99426439169815	9.24938908735172
C	2.47915930844661	18.44902467781421	9.65714302643755

C	2.35215909005445	16.95171169171050	9.58089268421931
C	1.40311260764766	16.23346572657291	10.28674487405066
C	1.32210645526839	14.85998922966283	10.10323741386769
C	2.19467887766142	14.23990445290948	9.22015852452482
C	3.12919760047632	15.00934087344364	8.55210347086817
C	4.03757026861092	20.19743079134170	8.80262328021011
C	5.31076675394441	20.25658880047220	8.00015821195953
C	6.04522845140060	21.41644285540189	7.82518177425674
C	7.18514218704238	21.38230894471593	7.03372315670081
C	7.56854384515568	20.18441419008523	6.44684750421036
C	6.80112180560177	19.05601805422958	6.66850634447638
C	4.74763677562946	18.66926026203662	10.60133754773213
C	5.82296962168326	17.63705470273414	10.42516299234801
C	6.72813694771911	17.35828858134500	11.43854833888257
C	7.70451296892333	16.40071096964495	11.22824897350868
C	7.75047243998049	15.74024420351114	10.00616449544161
C	6.82221464489508	16.05897412539545	9.03770198208739
H	2.14780164882574	18.83043388037791	10.62021567699940
H	1.85120608988078	18.89348892037804	8.88939452753474
H	0.74370875280070	16.74724210262439	10.96845641870503
H	0.59066080750950	14.28244853745775	10.64714224190507
H	2.16480490023270	13.17579880256747	9.05108031338045
H	3.83729414343966	14.58375604188064	7.85996748708048
H	4.02162463653583	20.96095423081198	9.57716982304569
H	3.18969811413862	20.37806080526731	8.14695624141954
H	5.72656857115852	22.32743381605713	8.30671461217422
H	7.76996562564989	22.27700026036273	6.88619325488105
H	8.45137885947272	20.11387607016307	5.83231318216496
H	7.05928120102644	18.09673347502604	6.24935420092278
H	5.20281951049860	19.62466523268326	10.85258427050994
H	4.12008601647224	18.39601633060357	11.44658072034137
H	6.65916012743914	17.89258946626203	12.37342807490977
H	8.41890713897315	16.17202598092350	12.00392909065446
H	8.49371218873705	14.98753174115135	9.79878260760115
H	6.80814533319490	15.58216644213149	8.07256947624866
O	3.05147236997030	18.22569837242435	6.75866359143641
N	3.07061200745351	18.61427037618100	5.51315018890770
C	2.09464501484382	19.49430019164928	5.12864780503183
C	2.04267814764125	19.95901167087039	3.85611216986884
C	2.99809221715017	19.52423760670180	2.89106336472507
C	3.97231866055177	18.58044409067029	3.32735742417872
C	3.99029023731831	18.14867954559342	4.61593508201315
H	1.39881133380857	19.77227807770010	5.89987351104410
H	1.25354947636787	20.64316461094251	3.60298404823926
H	4.72782892788257	18.20126100093523	2.66335533937516
H	4.69148931730062	17.43059869925314	5.00371695504648
N	2.98308014803762	19.98261792643542	1.63537769716326
C	2.08504932355647	21.05481044175885	1.20090942262831
C	3.86706053661785	19.45051636113142	0.59552855439079

H	1.89350865275314	21.75205178730593	2.00537636630665
H	1.15083197269585	20.61776716176356	0.84840410248435
H	2.56256843902517	21.57608618526180	0.37939856683841
H	4.80831717235665	20.00048589888674	0.60870944175804
H	3.38400627472993	19.60176203121360	-0.36246573190123
H	4.04930106640442	18.39417364570694	0.74302042822713
